# Giant honeybees (*Apis dorsata*) mob wasps away from the nest by directed visual patterns

**DOI:** 10.1007/s00114-014-1220-0

**Published:** 2014-08-29

**Authors:** Gerald Kastberger, Frank Weihmann, Martina Zierler, Thomas Hötzl

**Affiliations:** Department of Zoology, University Graz, Graz, Austria

**Keywords:** Giant honeybee, *Apis dorsata*, Shimmering, Defence waves, Repellence, Predatory wasp, Directional alignment

## Abstract

The open nesting behaviour of giant honeybees (*Apis dorsata*) accounts for the evolution of a series of defence strategies to protect the colonies from predation. In particular, the concerted action of shimmering behaviour is known to effectively confuse and repel predators. In shimmering, bees on the nest surface flip their abdomens in a highly coordinated manner to generate Mexican wave-like patterns. The paper documents a further-going capacity of this kind of collective defence: the visual patterns of shimmering waves align regarding their directional characteristics with the projected flight manoeuvres of the wasps when preying in front of the bees’ nest. The honeybees take here advantage of a threefold asymmetry intrinsic to the prey–predator interaction: (a) the visual patterns of shimmering turn faster than the wasps on their flight path, (b) they “follow” the wasps more persistently (up to 100 ms) than the wasps “follow” the shimmering patterns (up to 40 ms) and (c) the shimmering patterns align with the wasps’ flight in all directions at the same strength, whereas the wasps have some preference for horizontal correspondence. The findings give evidence that shimmering honeybees utilize directional alignment to enforce their repelling power against preying wasps. This phenomenon can be identified as *predator driving* which is generally associated with *mobbing* behaviour (particularly known in selfish herds of vertebrate species), which is, until now, not reported in insects.

**Electronic supplementary material:**

The online version of this article (doi:10.1007/s00114-014-1220-0) contains supplementary material, which is available to authorized users.

## Introduction

Giant honeybees have evolved shimmering behaviour for collective defence (Seeley et al. 1982; Kastberger et al. 2011a; Weihmann et al. 2012). Visual threats provoke patterns reminiscent of Mexican waves, which propagate with a characteristic velocity and in a controlled direction over the surface of a giant honeybee nest (Kastberger et al. 2012, 2013a, b). The first 200–300 ms of a shimmering wave form a flash-like visual signal with the capacity to repel a preying wasp (Kastberger et al. 2008, 2010; cf. Tan et al. 2012). Depending on the distance from the nest and the velocity of the threatening wasp, the shimmering waves vary in the repetition rate and in the recruitment of nest mates. Lastly, a startle reflex is initiated in the preying wasp, which makes her turn around and fly away from the bees’ nest (Kastberger et al. 2008).

However, shimmering is likely more sophisticated than just flashes of visual patterns which drive predatory wasps away from a giant honeybee colony (Kastberger et al. 2008, 2010; cf. Tan et al. 2012). On previous expeditions to India and Nepal (e.g. see the Movies in Kastberger et al. 2008, 2010), we suggested that the shimmering activities are tuned with the flight manoeuvres of the wasps in front of the bees’ nest, in particular in the directedness of wave propagation and of the repeated performance. This observation also led to the assumption that giant honeybees drive predatory wasps out of the nest range by aligning their collective response with the wasp’s flight. This *directed-shimmering-drives-wasps* hypothesis entails the control of rapidly changing external cues due to the flights of wasps on predation, based on a network of specialized bees on the nest surface (Schmelzer and Kastberger 2009) with the capacity to trigger hundreds of nest mates to align the spreading of their evoked visual patterns with the momentary direction of the wasp flight.

In this study, we show that shimmering behaviour does align, and even most of the time, with the flight of a wasp when preying in front of the bees’ nest and thus visible for surface bees (Online Resource 5–14/Movies 1–10). We bring evidence that the driving party in the mutual alignment is the bees in defence rather than the wasps threatening the bee nest. These findings support the *directed-shimmering-drives-wasps* hypothesis that the sophisticated control of the virtual directedness of the shimmering patterns efficiently helps the honeybees to increase the repelling power of the shimmering flashes (Kastberger et al. 2008) to *mob* preying wasps out of the range of the bees’ nest. Such kind of defence behaviour can be taken as a homologue to *mobbing* predators by selfish herds of vertebrate species (Hamilton 1971; Curio 1978; Arnold 2000; Alcock 2005; Chevalier 2013) but is, so far, unknown in the insect world.

## Material and methods

### Experimental site

The experiments were conducted with *Apis dorsata* nests in the village of Sauraha, at the border of the Chitwan National Park, Nepal. Preliminary experiments had been carried out in 2003 (Kastberger et al. 2008) and in 2009 (Kastberger et al. 2011b, 2012, 2013a, b; Weihmann et al. 2012), but in November 2010 a single nest was selected for the much broader in-depth investigation described in this paper. This approximately 3-week-old nest was attached to a hotel balcony (Fig. S1/Online Resource 1; Kastberger et al. 2011b, 2012, 2013b; Waddoup 2014), it had a hemispherical form of 85 × 60 cm (width × height) with a multi-layer cover comprising approximately 15,000 individuals.

### Displacement of an experimental wasp nest

The season of late November was selected for the experiments because at this time wasps are maximally abundant regarding colony size and numbers of nests. However, there was a paucity of free-flying wasps, even near giant honeybee nest aggregations, because Nepali farmers excessively exploit the wasp population due to their traditional use of wasp larvae as a supplementary protein source. They regularly burn the paper nests, barbecuing the wasp larvae and have thus reduced the population of wasps in Chitwan over a long period.

Therefore, the option of displacing wasp nests increased the chance for our experiments that single wasps approach the honeybee colony in its natural setting. We transferred a paper nest of *Vespa tropica* (individuals >100; brood cells >100) from 20 km away to the close vicinity of the experimental giant honeybee colony. After its relocation, the paper nest was encased with a curtain built of white and black linen. Utilizing the intrinsic positive phototaxis of forager wasps, we guided them through a tunnel of linen towards the experimental giant honeybee colony (Fig. S1/Online Resource 1) which they had to pass when departing or homing.

### Experimental sessions, video recording and image processing

**Fig. 1:**
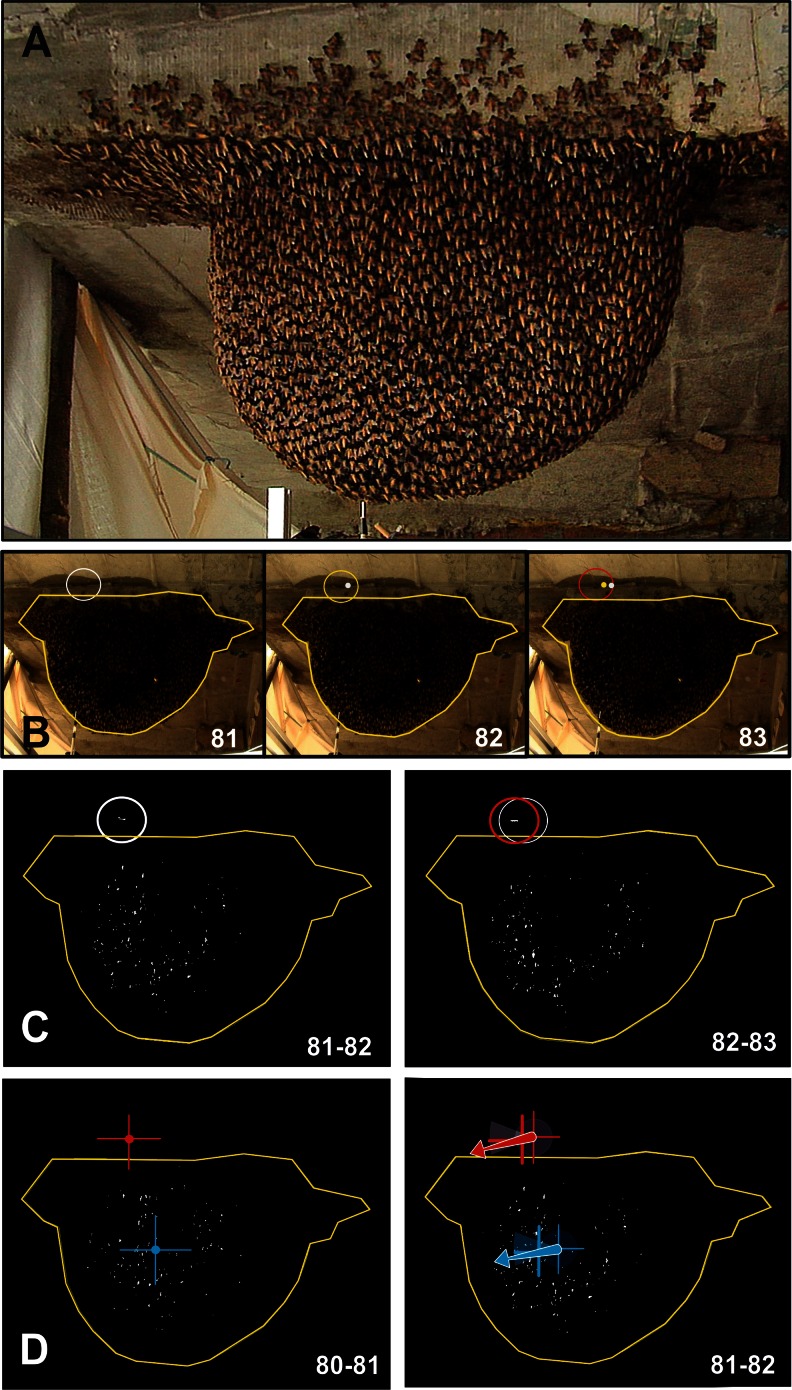
Directional coincidence of the movements of shimmering bees and of the preying wasp. **a** Experimental giant honeybee nest in quiescent state. **b** Three frames (ff 81–83; fps = 50 Hz) document a wasp on prey flying just above the bees’ nest and provoking shimmering waves; the wasp marked in the centre of the white circle (f 81) and in the centre of the yellow and red circles (ff 82,83) respectively; the *white* and *yellow points* mark the position of the wasp’s thorax in respective frames before; *yellow lines* give the nest contours. **c** Differential images (∆ff 81–82, ∆ff 82–83) displaying segmented motions (Δlum > 5) as *white spots*. Flight movement of the wasp was documented in the centre of the *white circle* (∆ff 81–82) and of the *red circle* (∆ff 82–83), respectively. **d** The cross-points quantify the centres of the momentary motions of wasp (*red*) and shimmering (*blue*) as evaluated in the differential image ∆ff 80–81; in image ∆ff 81–82, the *arrows* give the movement vectors as the connections of the cross points at ∆ff 80–81 and ∆ff 81–82

The experimental giant honeybee nest was filmed (e.g. Movie 1/Online Resource 5) with high definition video (Panasonic HVX 200) at 50 frames per second (fps) with a resolution of 1,280 × 720 pixels (px). The camera was placed from slightly below at a distance of 1.5 m to capture the bees’ nest (Figs. 1, S1/Online Resource 1) together with the wasps in front of it.

A film session of an interaction of preying wasps and shimmering honeybees started with the emergence of the wasp in the video image and ended with her disappearance (Movies 1–2/Online Resource 5–6). The sessions lasted 134.24 ± 17.03 frames corresponding to a footage duration of 2,684.80 ± 357.63 ms (n_ss_ = 50; fps = 50 Hz). The video films were formatted as jpg-sequences and processed by image analysis software (Image-Pro, Media Cybernetics) to detect wasp movements and shimmering activities of the honeybees. In total, 6,891 frames with wasps flying in front of the bees’ nest were collected (compare the sample Movies 1–10/Online Resource 5–6 comprising a single sequence over 179 ff ≡ 2,540 ms documenting different stages of the evaluation process).

### Assessment of shimmering movement

Shimmering behaviour happens exclusively in the surface layer of the bee curtain of a giant honeybee nest. In the quiescent state, the individuals hang with their heads up und their abdomens down (Kastberger et al. 2011a), but during shimmering they flip their abdomens upwards by more than 90° (Kastberger et al. 2011b) in a highly coordinated fashion which generates a Mexican wave-like process (Kastberger et al. 2012, 2013a, b).

Motion patterns of shimmering (sh) were detected by the differences in pixel luminance (lum_px_) in pairs of successive frames (f_i−1_, f_i_) referred to the focus frame at the time t_i_ (e.g. Movies 3–7, 9–10). To eliminate single-pixel noise (Kastberger et al. 2012, 2013a, b), the resulting Δ lum_px_ values (with Δ lum_px_ [f_i_] = lum_px_ [f_i_]–lum_px_ [f_i−1_]; Fig. 1b) were filtered by eroding and dilating functions and segmented into two classes: in the first class, no or minor change in luminance values (Δ lum_px_ ≤ 5) represented the “motionless” state; and in the second class, changes in luminance values of Δ lum_px_ > 5 signalled “movement”. This movement coding was used to calculate the coordinates of the horizontal position (x_sh_ [f_i_]) and of the vertical position (y_sh_ [f_i_]) of the momentary gravity point of the shimmering wave at the time t_i_ as explained in Eqs. 1a and 1b.

1a $$ {x}_{\mathrm{sh}}\left[{\mathrm{f}}_{\mathrm{i}}\right]\kern0.5em =\kern0.5em \varSigma\ {\mathrm{x}}_{\mathrm{m}}{\mathrm{a}}_{\mathrm{m}}\ /\varSigma\ {\mathrm{a}}_{\mathrm{m}} $$

1b $$ {\mathrm{y}}_{\mathrm{sh}}\left[{\mathrm{f}}_{\mathrm{i}}\right]\kern0.5em =\kern0.5em \varSigma\ {\mathrm{y}}_{\mathrm{m}}{\mathrm{a}}_{\mathrm{m}}/\varSigma\ {\mathrm{a}}_{\mathrm{m}} $$

whereas m_px_ = 1,2,..,n_px_ gives the number of segmented pixel areas at the focus frame f_i_, and a_m_ gives the pixel size of the spot m_px_.

Subsequently, the angular value (θ_sh_) of the direction of the shimmering wave was calculated (Eq. 2) on the basis of the positional changes of the gravity point (Eq. 1a and 1b) in the inter-frame interval Δt = t [f_i_]–t [f_i−1_] = 20 ms.

2 $$ \begin{array}{l}{\theta}_{\mathrm{sh}}\kern0.5em =\kern0.75em \mathrm{arc}\  \tan\ \left[\varDelta {\mathrm{x}}_{\mathrm{sh}}/\varDelta {\mathrm{y}}_{\mathrm{sh}}\right]\hfill \\ {}\mathrm{with}\ \varDelta {\mathrm{x}}_{\mathrm{sh}}\kern0.5em =\kern0.75em {\mathrm{x}}_{\mathrm{sh}\ }\left[{\mathrm{f}}_{\mathrm{i}}\right]\kern0.5em -\kern0.5em {\mathrm{x}}_{\mathrm{sh}\ }\left[{\mathrm{f}}_{\mathrm{i}-1}\right]\kern0.75em \mathrm{and}\ \varDelta {\mathrm{y}}_{\mathrm{sh}}\kern0.5em =\kern0.5em {\mathrm{y}}_{\mathrm{sh}\ }\left[{\mathrm{f}}_{\mathrm{i}}\right]\kern0.75em -\kern0.5em {\mathrm{y}}_{\mathrm{sh}\ }\left[{\mathrm{f}}_{\mathrm{i}-1}\right]\hfill \end{array} $$

### Assessment of the wasp movement

The wasps (w) typically hovered and scanned at an average distance of 50 cm from the honeybee nest (Kastberger et al. 2008). For every frame (f_i_), the positions of head and abdominal tip were interactively determined by mouse clicks (Fig. 1; Movies 2–10/Online Resource 6–14) allowing to assess the coordinates of the wasp thorax (horizontal, x_w_; vertical, y_w_) as their topological projections even under weak contrast. The momentary projected direction of the flight path (θ_w_) at the time point t [f_i_] was calculated by determining the displacement of the thorax point of the wasp per frame interval (Eq. 3).

3 $$ \begin{array}{l}{\theta}_{\mathrm{w}}\kern0.5em =\kern0.5em \mathrm{arc}\  \tan\ \left[\varDelta {\mathrm{x}}_{\mathrm{w}}/\varDelta {\mathrm{y}}_{\mathrm{w}}\right]\hfill \\ {}\mathrm{with}\ \varDelta {\mathrm{x}}_{\mathrm{w}}\kern0.5em ={\mathrm{x}}_{\mathrm{w}\ }\left[{\mathrm{f}}_{\mathrm{i}}\right] - {\mathrm{x}}_{\mathrm{w}\ }\left[{\mathrm{f}}_{\mathrm{i}-1}\right]\ \mathrm{and}\ \varDelta {\mathrm{y}}_{\mathrm{w}}\kern0.5em =\kern0.5em {\mathrm{y}}_{\mathrm{w}\ }\left[{\mathrm{f}}_{\mathrm{i}}\right] - {\mathrm{y}}_{\mathrm{w}\ }\left[{\mathrm{f}}_{\mathrm{i}-1}\right].\hfill \end{array} $$

### Interaction of shimmering wave propagation and wasp flight

*Assessment of mutual signalling between wasps and shimmering bees.* The behavioural context investigated here refers to the wasp as potential predator and to the shimmering bees as potential prey. In predator–prey interactions, signals are permanently produced by each of the parties eliciting responses in their counterparts. However, mutual signalling between wasps and shimmering bees happened at least in those time periods in which the wasps flew in front of the honeybees’ nest with the heads directed towards it and in which a shimmering wave spread over the nest surface. The two behavioural aspects with signal value considered here were the moving patterns of the flight path of the wasps (*w*; assessed as the positional changes of the wasp) and those of the shimmering waves (*sh*; assessed as the positional changes of the gravity centre of shimmering).

The interaction between wasps and shimmering bees was categorized by the similarity between the time series of the counterparts (*w* and *sh*) as a function of a time lag applied to one or the other of them. This method considers both counterparts at every time point in two roles: (a) as the *driving* signaller, which provokes the other party’s response, and (b) as the *driven* responder, which reacts to the signaller after a specific time lag. In the continuous sequence of interacting signals, the individual traits of both participants were separated by their roles as stimulating (*driving*) cue and as *driven* response to this cue by cross-correlating both time series (*w*, *sh*) using positive and negative time-lag filters. Therefore, cross-correlation of the two time series (*w*, *sh*) delivers a measure for the level of interaction between initiating cue and driven response (abbreviated as *cue → response*), whereby the sign of the time lag determined the state as *signaller* or *responder*. Consequently, two aspects of interaction can be considered: (a) the course of the gravity centre of shimmering as response to the flight course of the threatening wasp, abbreviated further on as *w → sh* and (b) the wasp’s flight course as response to the propagation direction of the shimmering wave (*sh → w*).

### Angular quantification of the directional alignment

**Fig. 2:**
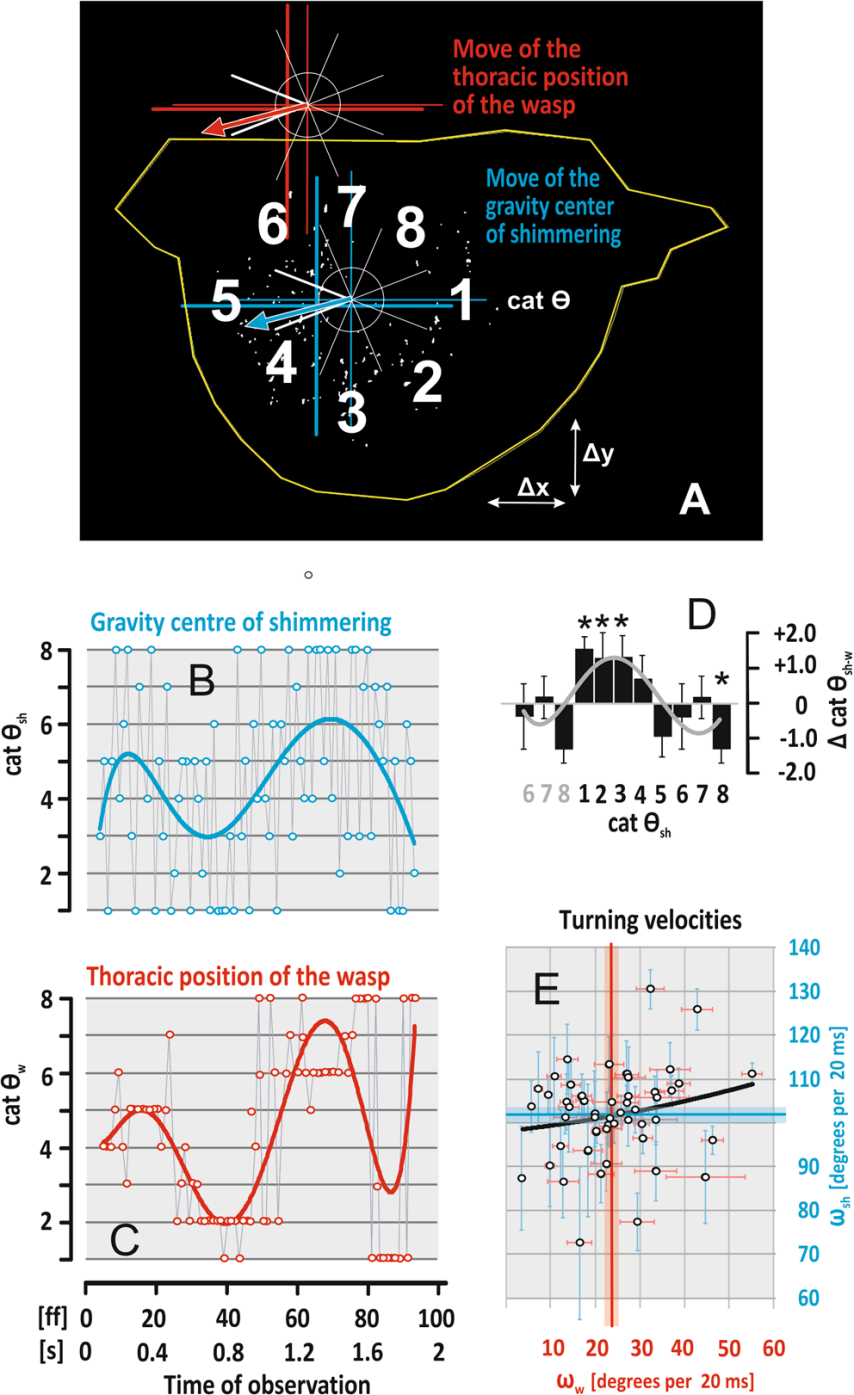
Characteristics of directional interactions between wasp flight and shimmering. **a** The movements of wasp (*w*: *red* colour coding) and shimmering (*sh*: *blue*) assessed over three sequential inter-frame intervals: in this example, the wasp flew above the attachment zone of the honeybee nest (cf. Fig. S1/Online Resource 1) to the left side of the image (*red arrow*; as calculated by the change in position in three sequential frames, cf. Fig. 1). The shimmering wave also propagated to the left (*blue arrow*; calculated by the change in position of the gravity centre, see; cf. Fig. 1). *White numbers* give the eight angular sectors (cat θ = [1]-[8]; cf. Fig. S2A/Online Resource 1) to classify the directions of *w*- and *sh*-movements. **b**–**c** A single observation session with two wave episodes covering 1.6 s (abscissa; time in frames [ff] and seconds [s]); ordinates, movement angles categorized by the angular sectors of cat θ (for definition, see panel A and Methods); curves are regression functions (*blue*, cat θ_sh_; *R* ^2^ = 0.1877; *red*, cat θ_w_; *R* ^2^ = 0.4153). **d** Differences (Δ cat θ_sh-w_, *black rectangles*, means; *vertical bars*, SEMs) between the courses of the shimmering waves (panel B) and that of the wasp (panel C) plotted against the direction of shimmering (cat θ_sh_); stars, significant deviations from zero (z test; n_ff_ = 81; *grey curve*, regression of the mean differences: *R* ^2^ = 0.5408). **e** Relation between turning velocities ((abscissa, ω_w_ = Δθ_w_/ Δt; ordinate, ω_sh_ in degrees per 20 ms; n_ss_ = 50 sessions; n_ff_ = 6,200 inter-frame intervals); means (*black open circles*) and SEMs (*coloured thin lines*) of the values of each of the sessions; *coloured triple lines* throughout the panel, overall means and SEMs; *black line*, linear regression function of mean values (*R* ^2^ = 0.0422)

For any time interval, the coincidence in movement direction of both traits (*w*, *sh*) was classified as *ipsi-directional* (I), *contra-directional* (C) or *intermediate* (im). Proper *ipsi-directional* coincidence is given for any time point t_i_ by equity of the angles (θ_sh_ = θ_w_,) and proper *contra-directional* coincidence is given by opposite angles (θ_sh_ = θ_w_ + 180°). For statistical purposes, we expanded this narrowly limited paradigm of coincidence by the range of Δ θ_A_ = 45° (Fig. S2A/Online Resource 2) to assign *ipsi-directionality* (abbreviated further as DIR_I_) with θ_sh_ = θ_w_ ± Δ θ_A_ and *contra-directionality* (DIR_C_) with θ_sh_ = θ_w_ + 180° ± Δ θ_A_ (Fig. S2B/Online Resource 2). The missing intervals between *ipsi-* and *contra-directionality* are classed as *intermediate* alignment (DIR_im_: θ_sh_ = θ_wasp_ ± 90° ± Δθ_A_/2). For simplification, we categorized the *alignment* conditions (DIR_I_, DIR_C_ and DIR_im_; defined by Eqs. 4a, 4b and 4c) by eight angular sectors (cat θ = [1–8]) comprising 360° (see the sketches in Figs. 2, S2/Online Resource 2).

4a $$ {\mathrm{DIR}}_{\mathrm{I}}:\ \varDelta\ \mathrm{cat}\ {\theta}_{\left[\mathrm{w},\mathrm{sh}\right]}\equiv \left[+1\right]\ \mathrm{or}\ \left[0\right]\ \mathrm{or}\ \left[-1\right] $$

4b $$ {\mathrm{DIR}}_{\mathrm{im}}:\ \varDelta\ \mathrm{cat}\ {\theta}_{\left[\mathrm{w},\mathrm{sh}\right]}\equiv \left[+2\right]\ \mathrm{or}\ \left[-2\right] $$

4c $$ {\mathrm{DIR}}_{\mathrm{C}}:\ \varDelta\ \mathrm{cat}\ {\theta}_{\left[\mathrm{w},\mathrm{sh}\right]}\equiv \left[4\right]\ \mathrm{or}\ \left[+3\right]\ \mathrm{or}\ \left[-3\right] $$

For the aspect of *w → sh*, the difference in alignment is calculated by Δ cat θ (*w → sh*) = cat θ _[sh]_–cat θ _[w]_ and for the aspect of *sh → w* this difference is Δ cat θ (*sh → w*) = cat θ _[w]_–cat θ _[sh]_ (as exemplified in Fig. S2A/Online Resource 2).

### Comparison of empirical data with a mathematical movement heading model

Per definition, two parties are randomly aligned if both movements equal to a *random walk* (Einstein 1926), the mathematical formalization of a path that consists of a succession of random steps. Movements which differ from *random walk* are supposed to arise from reactions, intentions or decisions. The *split-component method* was used to detect coincidence in the directionality of the time series *w* and *sh* for the movement components (Δx and Δy) per inter-frame interval. Two modes of alignment can be distinguished: (a) *ipsi-directionality* (abbreviated as x_I_ or y_I_), if the movement components of both counterparts (Δx_w_, Δx_sh_ and Δy_w_, Δy_sh_) changed within a single inter-frame interval with the same sign (e.g. referring to the horizontal components: [Δx_sh_ > 0, Δx_w_ > 0] or [Δx_sh_ ≤ 0, Δx_w_ ≤ 0]). (b) *Contra-directionality* (abbreviated as x_C_ or y_C_), if the movement components differed in their signs (e.g. referring to the horizontal component: [Δx_sh_ > 0, Δx_w_ ≤ 0] or [Δx_sh_ ≤ 0, Δx_w_ > 0]). Each step of interaction represents one pair of alignment defined by the movement steps of both counterparts ([Δx_sh_, Δy_sh_] and [Δx_w_, Δy_w_]). This allows distinguishing *ipsi-directional* ([x_I_, y_I_], abbreviated further on as DIR_1_), two *intermediate* alignment conditions ([x_C_, y_I_]: DIR_2_; [x_I_, y_C_]: DIR_3_) and *contra-directional* ([x_C_, y_C_]: DIR_4_).

In this paper, we introduce a mathematical movement heading model which uses this split-component method on the basis of a doubly randomized algorithm (with 0.00 ≤ rnd (q) ≤ 1.00) to separately assign *value* (=q_value_) and *sign* (=q_sign_) of each of the directional components for every step of movement of both parties in interaction. A path of movement can be described by its heading factor *h* which refers to the statistical difference of the probabilities (P) between *positive* directions (Δx > 0, directed to the right in the image; Δy > 0, directed to the top in the image) and *negative* directions (Δx < 0, Δy < 0) over time according to Eqs. 5a and 5b.

5a $$ {\mathrm{h}}_{\mathrm{x}}=\mathrm{P}\ \left(\varDelta \mathrm{x}>0\right)\kern0.5em -\kern0.5em \mathrm{P}\ \left(\varDelta \mathrm{x}<0\right) $$

5b $$ {\mathrm{h}}_{\mathrm{y}}\kern0.5em =\kern0.5em \mathrm{P}\ \left(\varDelta \mathrm{y}>0\right)\kern0.5em -\kern0.5em \mathrm{P}\ \left(\varDelta \mathrm{y}<0\right) $$

Given the position of both parties (x_0_ and y_0_) at the time t_0_ and the *heading* factors for both directional components, the *value* of the subsequent movement step was defined by Δx = rnd [Δx_value_] and Δy = rnd [Δy_value_]. Its *sign* was determined by initializing functions (sign [Δx] = rnd [Δx_sign_]; sign [Δy] = rnd [Δy_sign_]) and a threshold algorithm regarding the time interval (Δt = t_i_−t_0_) which defines the sign of both directional components (Δc as Δx or Δy) as positive for rnd [Δc_sign_] < h_c_ and negative for rnd [Δc_sign_] > h_c_. In the model, the theoretical position of both parties at the time point t_i_ was calculated by integration of the *x*- and *y*-components according to Eq. 6a and Eq. 6b.

6a $$ {\mathrm{x}}_{\mathrm{i}}\kern0.5em =\kern0.5em {\mathrm{x}}_0\kern0.5em +\kern0.5em \mathrm{sign}\ \left(\mathrm{rnd}\ \left[\varDelta {\mathrm{x}}_{\mathrm{sign}}\right]\right)\ *\ \mathrm{rnd}\ \left[\varDelta {\mathrm{x}}_{\mathrm{value}}\right] $$

6b $$ {\mathrm{y}}_{\mathrm{i}}\kern0.5em =\kern0.5em {\mathrm{y}}_0\kern0.5em +\kern0.5em \mathrm{sign}\ \left(\mathrm{rnd}\ \left[\varDelta {\mathrm{y}}_{\mathrm{sign}}\right]\right)\ *\ \mathrm{rnd}\ \left[\varDelta {\mathrm{y}}_{\mathrm{value}}\right] $$

For explanation, the heading factors h_x_ and h_y_ are zero under *random walk* condition, but bigger heading values will result in *walking* paths as displayed in Fig. S3A/Online Resource 3. For instance, a heading factor of h_x_ = +0.50 means that the horizontal movement component (Δx) was positive in 75% of the cases (represented here by the time steps Δt) and it was negative in the complement of 25% of cases.

Directional alignment of two *walking* parties can be described by the momentary values of their headings. Random alignment is given if the headings of each of them are zero (h_x_[*w*] = h_x_[*sh*] = h_y_[*w*] = h_y_[*sh*] = 0). In terms of the normalized distributions of alignment, these *random walk* conditions result in uniform rates of the alignment components (r [DIR_1-4_] = 0.25). Variation of the heading factors of the two parties, reflected in the probability histograms in Fig. S3B/Online Resource 3, can be extended into the lookup table as displayed in Fig. S3C/Online Resource 3.

## Results

### Directional alignment of shimmering waves with the flight paths of preying wasps

When wasps prey in front of a giant honeybee nest, they provoke shimmering waves (Kastberger et al. 2008). Thus, both counterparts (*w* and *sh*) display moving patterns which happen, topologically seen, on roughly parallel vertical planes (Fig. 1): the shimmering waves propagate over the nest surface layer and the preying wasps fly at an average distance of 50 cm (Kastberger et al. 2008) from the nest surface. The frame-wise analysis of the projected movement angles of both parties (θ_w_, θ_sh_; see Figs. 2a, S2A/Online Resource 2) demonstrates strong coupling of both directional vectors. A joint course can be documented even for a single session of interaction, illustrated by the regression functions (Fig. 2b–c) and confirmed by the distribution of the differential angles (Δ cat θ_w-sh_ in Fig. 2d). Both movements differed weakly (-45° ≥ Δcat θ_sh-w_ ≥ +45°) and only in four from eight angular cat θ_sh_ sectors [1-3, 8].

This correspondence in the movements of wasp and shimmering is further illustrated in the film episode (Movies 1–10) in which the first shimmering wave was elicited when the wasp came into the visual range of the honeybee colony. Then, the wasp flew from the left side to the mid of the image near the lower nest rim while the shimmering wave propagated from here in up-right direction (which is roughly parallel to the wasp flight path). Possibly intimidated by the wave (Kastberger et al. 2008), the wasp responded by flying downwards and subsequently by turning around to fly back to the left side of the image and leaving the nest range. During this flight manoeuvre, a further shimmering wave was elicited which again propagated roughly in the same direction as the wasp path (from the lower rim to the up-left nest side).

In total, the two counterparts differed significantly in their turning velocities (ω = Δθ/Δt). The wasp produced turning velocities of ω_w_ = 23.87 ± 1.57 [degrees per 20 ms] while the shimmering process was more than four times as fast (ω_sh_ = 101.45 ± 1.48° per 20 ms) and, nonetheless, independent from the wasp’s movement direction (*R* ^2^ = 0.0422; *P* = 0.2040, Pearson test; n_ss_ = 50 sessions; n_ff_ = 6,891 inter-frame intervals; Fig. 2e).

**Fig. 3:**
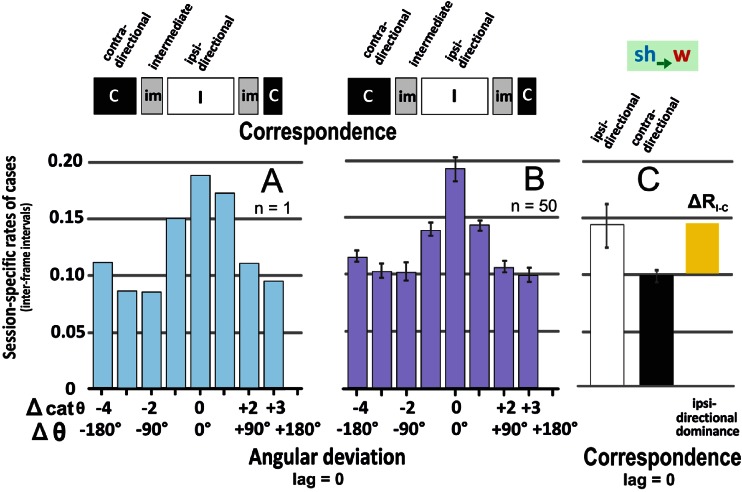
Angular deviations between wasp and shimmering wave (*sh → w*, lag = 0). Session-specific rates of cases displaying angular deviation between wasp and shimmering. Abscissa, eight classes of angular deviation scaled in Δ cat θ and Δ θ (for definition, see Figs. 1a, S2/Online Resource 2); ordinate, relative number of cases (≡inter-frame intervals of 20 ms) per angular deviation class; The value 1.0 refers to the total of cases per session. **a** The distribution of the rate of cases exemplified for the session 43 (*blue columns*). **b** Means (*violet columns*) and SEMs (*grey vertical bars*) of all sessions (n_ss_ = 50). The graphs above the histograms define the angular sectors for *ipsi-directional* (code I; *white colour* code), *intermediate* (im; *grey*) and *contra-directional* (C; *black*) correspondence between the directions of the wasp’s flight (*w*) and of the movement of the gravity point of shimmering (*sh*) per time step. **c** The difference between the session-specific mean rates (R) of *ipsi*- *directional* (*white column*) and *contra-directional* (*black column*) cases of alignment, termed as *ipsi-directional dominance* (D_id_ ≡ Δ R_I-C_; *yellow column*)

**Fig. 4:**
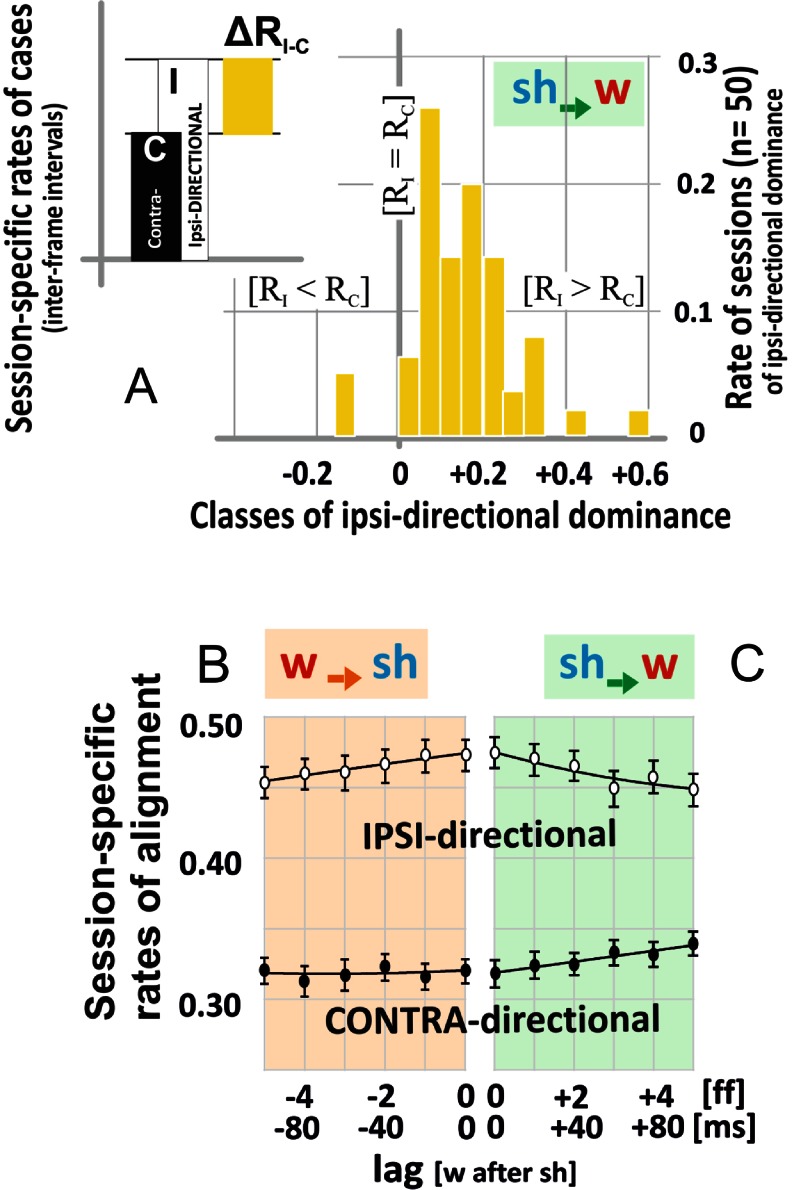
Characteristics of alignment between wasps and honeybees (*w → sh*, *sh → w*). **a** Histogram of the cases of *ipsi-directional dominance* (D_id_, n_ss_ = 50); abscissa, dominance classes of *ipsi*- (I) and *contra*- (C) -*directional* alignment (∆R_I-C_; *sh → w*; lag = 0) with the definitions: class [0], equity conditions [R_I_ = R_C_]; class [+1], [R_I_ = 1, R_C_ = 0]; class [-1], [R_I_ = 0, R_C_ = 1]. Ordinate, rate of sessions of *ipsi-directional* dominance D_id_ (*yellow*). **b**-**c** Session-specific rates (ordinate) of alignment, under both modes of interaction (*sh → w*, *w → sh*), whereby the total of cases sums up to the value of 1.0 per session (ΣR = R_I_ *+* R_im_ *+* R_C_ = 1); abscissas give the latency selected (lag [*sh → w*] ≡ lag [*w* after *sh*]) in frames [ff] and milliseconds [ms]; data volume: n_ff_ = 6.891 inter-frame intervals; n_ss_ = 50 sessions

Coincidence in the movement directions of both parties (*w* and *sh*) can be particularly demonstrated if their alignment rates are viewed synchronously (Fig. 3). The rates of *ipsi-directional* alignment (R_I_ = 0.1920 ± 0.0121, at Δ cat θ_w-sh_ = [0]) were significantly higher than those of *contra-directional* alignment (R_C_ = 0.1157 ± 0.0050, at Δ cat θ_w-sh_ = [-4]; *P* < < 0.001; *t* test). Similar results are achieved for the broader comparison, with the expansion of *ipsi-* and *contra-directionality* by combinations of angular sectors (see the logic schemes in Fig. 3 and Materials and methods). The positive factor R_I_/R_C_ under synchronous conditions (Fig. 3c) displays here the phenomenon of *ipsi-directional dominance* (D_id_) which was traced in singular sessions (e.g. Fig. 3a; n_ff_ = 191 ff. *P* < 0.001, *χ* ^2^ test) and proved for the entirety of sessions investigated (ΔR_I-C_ > 0: *P* < 0.001, *t* test; n_ss_ = 50 sessions; n_ff_ = 6,891 inter-frame intervals; Fig. 3b,c). Figure 4a shows the D_id_ histogram regarding dominance classes for the example of the wasp as responder (*sh → w*); it refers to synchronous assessment (lag = 0) of all sessions (n_ss_ = 50) observed.

### Ipsi- and contra-directional alignment of shimmering waves with the wasps’ flight paths under time-shift conditions

The streams of behaviours as documented in the Movies 1–10 result from the continuous interactions of both parties upon each other. We isolated the reactions of each party from the continuum of mutual signals (*w-sh-w-sh*-…) by utilizing the principle that any reaction happens after any stimulus but with delay. Fast flyers, such as wasps and bees, display latency periods not shorter than 20 ms. Therefore, a time window of up to five frames (≡100 ms) will be sufficiently long to trace any responsiveness of each of the parties expressed by their motions. For the graphical summarization in Fig. 4b and c, the wasps’ responses (*sh → w*) are displayed by positive time shifts (lag [*w* after *sh*] > 0), and the responses of the bees (*w → sh*) are displayed by negative time shifts (lag [*w* after *sh*] < 0). The results document *ipsi-directional dominance* (D_id_: R_I_ > R_C_) in both aspects of interactions under all time-shift conditions investigated (*P* < 0.001, t test; n_ss_ = 50) whereas D_id_ decreased slightly but significantly with increasing absolute lags.

### Asymmetry in ipsi-directional dominance

**Fig. 5:**
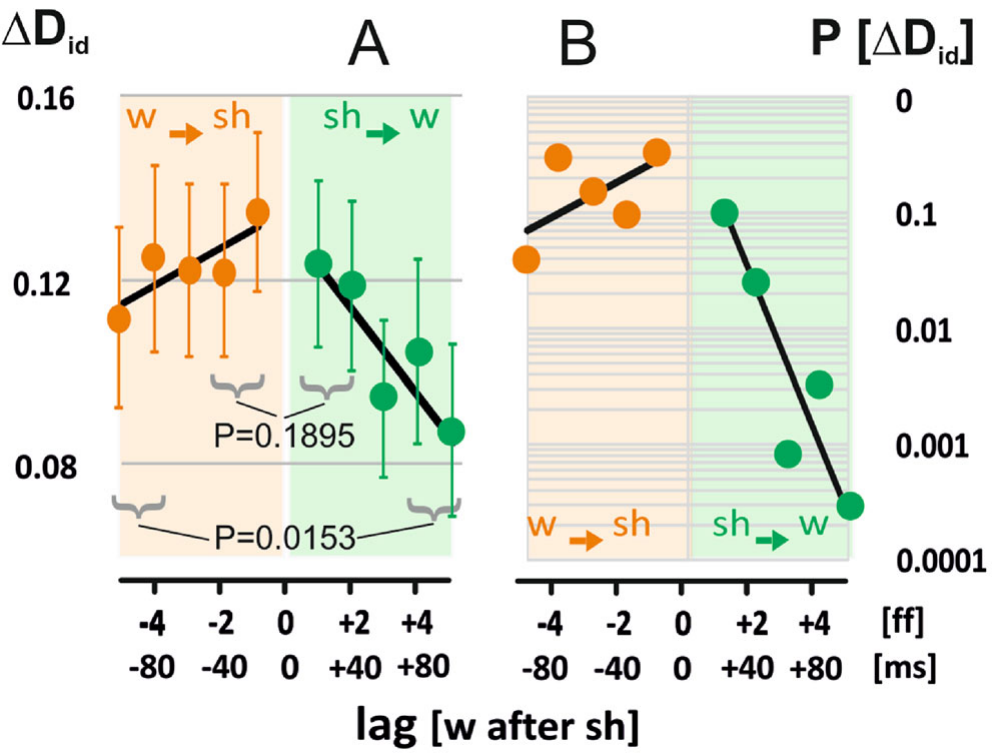
Asymmetry of *ipsi-directional* dominance (D_id_) between wasp and shimmering (*w → sh*, *sh → w*). **a** Session-specific differences in ipsi-directional dominance with the session-specific synchrony conditions as reference (∆D_id_ = {∆R_I-C_ [│lag│ > 0]–ref D} with ref D = ∆R_I-C_ [lag = 0]; for further information, see Figs. 3–4). Asymmetry between both aspects (*brown*, *w → sh*; *green*, *sh → w*) is proved at higher lags (│lag│ = 0–1: *P* = 0.1895; │lag│ = 4-5: *P* = 0.0153, *t* test; n_ss_ = 50). *Circles*, means; *vertical bars*, SEMs. **b** Significance values (*t* test) of the differences of D_id_ values (n_ss_ = 50) between lagged and synchronous conditions; ordinate P (∆D_id_) = *P* {∆R_I-C_ [│lag│ > 0]–ref D}, in both aspects of interactions. Abscissa: lag [*w* after *sh*]) scaled in ff and ms (cf. Figs. 3–4)

We compared the values of D_id_ [│lag│ > 0] with those under synchronous assessment as reference (ref D = D_id_ [lag = 0]), separately for every session (n_ss_ = 50) for wasps and shimmering bees. The differences of the D_id_ values between both lag conditions (ΔD_id_ = D_id_ [│lag│ > 0]–ref D) decreased with the absolute lag value (Fig. 5), but, which is important, differently strongly in the two aspects of interaction: for shimmering bees as *responders* (*w → sh*), the ΔD_id_ values differed slightly from the reference, whereas for wasps as *responders* (*sh → w*) the ΔD_id_ values decreased much stronger with increasing time shifts (Fig. 5a). Consequently, at longer time shifts (│lag│ > 3) shimmering bees as *responders* had larger ΔD_id_ values (*P* = 0.0153, *t* test; Fig. 5a) and their rates P (∆D_id_) differed more strongly than with wasps as *responders*.

In a second independent analysis (Fig. S4/Online Resource 4), we counted the signs of the ΔD_id_ values of each session to classify two categories of *ipsi-directional* dominance (see sketch in Fig. S4A/Online Resource 4). For ΔD_id_ > 0, the alignment response exceeded the reference (ref D) displaying the *supra-reference* status of *ipsi-directional dominance* (supra-D_id_), whereas for ΔD_id_ < 0 the alignment response was classified as *sub-reference* status (sub-D_id_). The rates of *supra*-D_id_ traits (Fig. S4B/Online Resource 4; *P* = 0.0135, paired *t* test; n_lag_ = 5) and the magnitudes of *supra*-D_id_ values at bigger latencies (│lag│ > 2; *P* = 0.0020, *t* test; n_*sh→w*_ = 49, n_*w→sh*_ = 56; Fig. S4C/Online Resource 4) were higher in shimmering bees as *responders* than in preying wasps as *responders*. Conversely, the magnitudes of *sub*-D_id_ rates (Fig. S4C/Online Resource 4) decreased with absolute latencies in both aspects of interaction.

Both findings (Figs. 5, S4/Online Resource 4) demonstrate that shimmering bees have the capacity to align their collective response with the wasp’s flight direction significantly longer, at least up to 100 ms, than the wasps are able to align their flight paths to the propagation direction of the shimmering waves. This is proved by the findings that the wasps significantly reduced both magnitude and rate of ΔD_id_ (*sh → w*: Fig. 5) already 40 ms past the onset of the driving cue, whereas shimmering bees even increased the magnitude of supra-D_id_ values at time shifts of >40 ms (*w → sh*: │lag│ > 2; Fig. S4C/Online Resource 4).

### Dependence of alignment of shimmering waves with preying wasps on topological relations

**Fig. 6:**
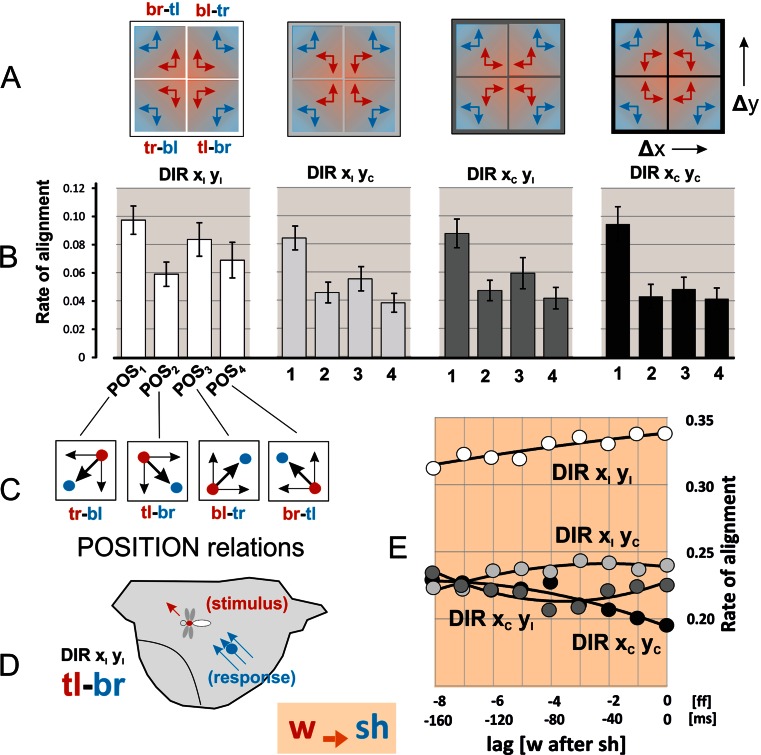
Directional alignment (*w → sh*) as assessed with the split-component method. This approach distinguishes four directional conditions of the wasp (coded *red*) and the gravity centre of the shimmering wave (coded *blue*). **a** The sketches explain the pairs of positional deviations (Δx and Δy) given for the four categories of alignments with *ipsi-directional* (code I) and *contra-directional* (code C) dispositions (as displayed in panel B). **b** Ordinate, alignment rates (DIR x_I_, y_I_, *white bars* in the histograms; DIR x_I_, y_C_, *bright grey*; DIR x_C_, y_I_, *dark grey*; DIR x_C_, y_C_, *black*) sorted after four positional relations (abscissa POS_1–4_, see panel C) summed up to the rate value of 1.0 for each of the sessions investigated (n_ss_ = 50); *Histogram bars*, arithmetical means; *vertical lines*, SEMs; the empirical data were exemplified for latency conditions of lag = 1. **c** Four positional relations: POS_1_: tr-bl; POS_2_: tl-br; POS_3_: bl-tr; POS_4_: br-tl; with *t* top; *r* right; *b* bottom; *l* left). **d** The specific constellation tl-br, where the wasp as *stimulus* was positioned in the given frame at the top-left (*red*: tl) of the gravity centre of the shimmering wave as *responder*. Conversely, the gravity centre of the shimmering wave was positioned at the bottom-right (*blue*: br) of the wasp. **e** Summarization of alignment rates regarding the four directional relations (DIR_1–4_): ordinate, the rate of alignment as defined in panel B; *circles*, medians of alignment rates regarding POS_1–4_, normalized to 1.0. Abscissa, latency in frames [ff] and milliseconds [ms] between wasp flight as *stimulus* and shimmering wave as *response*; regression polynomials: DIR x_I_ y_I_, *R* ^2^ = 0.7927; DIR x_I_ y_C_, 0.8160; DIR x_C_ y_I_, 0.7577; DIR x_C_ y_C_, 0.9187

**Fig. 7:**
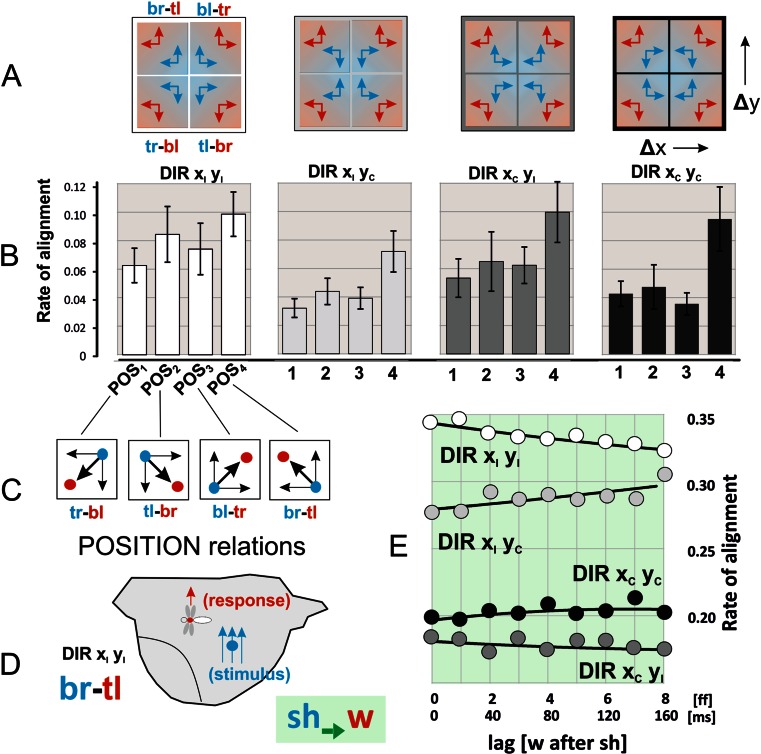
Directional alignment (*sh → w*) as assessed by the split-component method. The sketch in panel D displays the sample constellation br-tl when the wasp positioned in the given frame at the top-left (*red*: tl) of the gravity centre of the shimmering wave (*blue*: br) was considered as *responding* to the shimmering wave as *stimulus*. **e** Regression polynomials: DIR x_I_ y_I_, *R* ^2^ = 0.8564; DIR x_I_ y_C_, 0.5698; DIR x_C_ y_I_, 0.2323; DIR x_C_ y_C_, 0.3841). For further information, see Fig. 6

A typical *A. dorsata* nest is shaped alike a hemispherical plate and measures mostly more than 1 m in the horizontal span. It also has specialized regions, most of them peripheral to the mouth region, where shimmering waves are generally initiated (Schmelzer and Kastberger 2009). A wasp usually preys around the bees’ nest by hovering or scanning at an average distance of 50 cm (Kastberger et al. 2008), also below and above the nest, possibly by favouring certain regions of it. Therefore, it makes sense to include the relations between the positions of the wasp and of the gravity centres of the shimmering waves at every time step of interaction, from both perspectives (*w → sh* and *sh → w*). For that, four positional conditions were distinguished (POS_1–4_: Figs. 6c and 7c) in which the positional centre of one party was at the right, left, top or bottom side of that of the other party.

The histograms (Figs. 6b and 7b) exemplify the aspect of shimmering bees and wasps as *responders* for the time shift of 20 ms (│lag│ = 1). The alignment rates were sorted into four directional (DIR_1–4_) and four positional (POS_1-4_) classes while for each experimental session (n_ss_ = 50) the total rate of inter-frame intervals was normalized as 1.0. The findings display preference of the wasps for specific positions at the nest, which probably results from site properties of the honeybee nest (and which would certainly differ with other nests). For the shimmering bees as *responders* (*w → sh*; Fig. 6b), most of the cases of alignment happened, irrespective of *ipsi*- or *contra-directionality*, at the positional relation POS_1_ (≡*tr-bl*), in which the wasp as stimulus was *top-right* (*tr*) to the gravity centre of the shimmering wave. Cross-referencing with the other aspect of interaction (*sh → w*; Fig. 7b) confirms that the positional preference of the wasp as *responder* was given at POS_4_ (≡*br-tl*).

### Directional preferences for alignment

The *defence hypothesis of shimmering* has been proved for the aspect that shimmering waves have the potential to repel threatening wasps away from the nest (Kastberger et al. 2008). If directional alignment of shimmering waves with the wasp’s flight favours this repelling ability, shimmering waves should “follow” the wasp with the same diligence in any direction on the vertical plane of the nest surface (see Movies 1–10). With other words, the bees should not prefer in their alignment ability any flight direction of the wasp. Conversely, it can be expected that the wasp is neither benefitted by the direction of shimmering nor by her own alignment reaction to shimmering waves. Therefore, the wasp could likely be influenced in her responsiveness by secondary features such as site attributes of the bees’ nest (as actually demonstrated in the Figs. 6b and 7b).

Using the split-component method (see Material and methods), the attendances for directional alignment of shimmering bees and wasps in their roles as *responders* to the other party’s signals (*sh → w*, *w → sh*) were distinguished into four DIR_1–4_ classes (Figs. 6e and 7e). Both parties mainly align *ipsi-directionally* (DIR x_I_ y_I_) whereby the rates decreased with increasing latency periods. The bees as *responders* (*w → sh*) aligned at a lower level (Fig. 6e) under partial and full *contra-directionality* (DIR x_C_y_I_, DIR x_I_ y_C_, DIR x_C_ y_C_). This gives evidence that the bees responded to all flight directions of the wasp with the same strength of alignment, conforming to the predictions of the *defence hypothesis* of shimmering. Conversely, the wasps as *responders* (*sh → w*) were more prone to horizontal than to vertical alignment with shimmering. This is displayed by the DIR x_I_ y_C_ graph in Fig. 7e which is positioned between proper *ipsi-directionality* (DIR x_I_ y_I_) and x-based *contra-directionality* (DIR x_C_ y_I_, DIR x_C_ y_C_).

### Estimation of the empirical headings

We compared the case-specific proportions of alignment values (r [DIR_1-4_], see Figs. 6 and 7) with those of the mathematical heading model (Fig. S3/Online Resource 3) according to the best-match (max-*χ* ^2^) criterion. In the simplest assumption that both parties (*w* and *sh*) equal in the headings (h_x,y_ [*sh*] = h_x,y_ [*w*]) the best match will result at a heading of *h* = 0.34 (coded by green colour in Fig. S3A,B,E/Online Resource 3).

For the best match with the empirical data, the two pairs of headings (h_x,y_ [*sh*], h_x,y_ [*w*]) were permutated stepwise. Figure S3D/Online Resource 3 illustrates here the last step of such a search for a selected proportion of headings (h_x_[w] = 0.59, h_y_[w] = 0.67, h_x_[sh] = 0.62), for which the missing vertical heading component was determined at the maximum of the respective regression polynomial P[*χ* ^2^] = f (h_y_[sh]) with h_y_[sh] = +0.154. Otherwise, the best-match permutation for the equity conditions of all partial headings (h_x_ [*sh*] = h_y_ [*sh*]; h_x_ [*w*] = h_y_ [*w*]) can be assessed according to the look-up table of Fig.S3/Online Resource 3 (green arrows). That way, multiple runs of the model led to the determination of the heading components of the empirical wasp’s flights with h_x_ [*w*] = 0.18 and h_y_ [*w*] = 0.34 and of the associated shimmering waves with h_x_ [*sh*] = 0.24 and h_y_ [*sh*] = 0.16.

These findings show that the wasp slightly emphasized the vertical heading whereas the shimmering responses were more uniform herein. Interestingly, they also may explain the conundrum of the *ipsi-directional dominance* (D_id_) in alignment under synchronous assessment conditions (Figs. 3 and 4b,c) because such alignment conditions (displayed by the green histograms in Fig. S3B/Online Resource 3, in comparison with the histograms in Figs. 6b and 7b) differed significantly from the *random-walk* process. Therefore, it is plausible to assume that the phenomenon of *ipsi-directional dominance* does result from an alternative process, most likely from the interference of the continuous, mutual and asymmetric interactions of the parties, which themselves switch repetitively between *signaller* and *responder* roles.

## Discussion

### The multiple goals of defence of shimmering

The wave-like shimmering in giant honeybees (Seeley et al. 1982; Oldroyd and Wongsiri 2006; Woyke et al. 2006; Kastberger et al. 2012, 2013a, b) is a collective action of the bees on the nest surface with the potential to repel predatory wasps (e.g. the autochthon species of *Vespa orientalis*, *V. tropica*, *Vespa mandarinia*). Such wasps usually hover or scan in front of the bees’ nest, preying on the stationary curtain bees or ambushing homing or departing forager bees in flight (Kastberger et al. 2008, 2011a). It is important to note here that the interactions between shimmering bees and preying wasps occur exclusively in the visual domain and that the repelling impact of shimmering is connected only with its flash-like rise in the *ascending* phase of the shimmering process (when the synchronously active cohort of bees increases in size within the period of 200-300 ms; see Kastberger et al. 2008, 2013b). This kind of visual flash elicits an avoidance response in the wasp addressee, making it fly away from the bees’ nest. However, as soon as the synchronously or serially shimmering-active cohort became smaller, at about 300 ms after the onset of the wave, the wasp resumes her preying activity (Kastberger et al. 2008).

Shimmering behaviour, however, provides additional features to dispel wasps from the range of the bees’ nest. If the wasp comes nearer to the nest or if she increases her flight speed in passing by the nest, the repetition rate of the shimmering waves is increased (Kastberger et al. 2008). If the threatening wasp eventually touches the nest, small local waves are produced which efficiently confuse the wasp and hamper its further effort to prey directly from the nest surface (Kastberger et al. 2008). If, nevertheless, a wasp is still in the touching position with the nest to capture a bee from the nest surface, curtain bees immediately draw her into the nest heat-balling her to death (Kastberger and Stachl 2003; Kastberger et al. 2011b) alike in other *Apis* species (*Apis cerana*: Ono et al. 1987, 1995; Ken et al. 2005; *Apis mellifera*; Tan et al. 2007; *Apis florea*; Seeley et al. 1982). All these facets of collective defence make it practically impossible for a wasp to concentrate on distinct spots and to capture a bee directly from the surface of a giant honeybee nest.

In this paper, a further defence goal of shimmering has been addressed, which may be counted among the most sophisticated traits in collective defence in insects (Johnstone 1997; Smith and Harper 2003; Alcock 2005). It is the specific ability of shimmering-active surface bees to couple the direction of their collectively produced wave with the momentary direction of the flight path of a threatening wasp (Movies 1–10). Importantly, this alignment behaviour initiated by the bees makes the wasp in turn follow the shimmering wave. This capacity of the shimmering-active surface bees seems to be an example of *predator driving* (termed as the *mobbing* analogue of *prey driving*; see Coppinger 2001; Hayward et al. 2011), which is documented in context with *mobbing* behaviour in vertebrates (Curio 1978; Dominey 1983; Arnold 2000; Osthreiher 2003). Mobbing behaviours have been reported from individuals of species of potential prey (Curio 1978; Alcock 2005), that cooperatively attack or harass unwanted intruders (cf. Chevalier 2013), usually to protect their offspring or to drive them away from a food source. In particular in birds, mobbing behaviour includes a series of displays such as flying about, dive bombing, loud squawking, defecating on the intruder and even summoning nearby individuals to cooperate in the attack by producing mobbing calls (Leger and Carroll 1981; Alcock 2005).

The findings in *A. dorsata* (Kastberger et al. 2008 and this paper), however, leave it still unclear whether shimmering in honeybees conforms to any of the latter mobbing theories. What is known up to now is that shimmering giant honeybees, as potential prey, is not likely to warn the intruders away from the nest by signalling physical fitness (which, however, has been proposed for the homologous behaviour in *A. cerana*; Tan et al. 2012). We see strong evidence that the flash-like shimmering patterns actually force the preying wasp to escape. The bees utilize particularly here startle reactions of the wasp as response to the *ascending* recruitment of shimmering participants in the first 300 ms of a wave (Kastberger et al. 2008, 2013b). But in a second defence line, shimmering also provides the phenomenon of directional alignment between the honeybees as prey and wasps as predators, which can be well compared to defence actions such as an African buffalo herd’s when confronting a lion, of lining up and attacking it (Alcock 2005; Chevalier 2013). The behaviours in both scenarios (shimmering bees vs. wasp and buffalo herd vs. lion) have at least the same consequence that the counterparts, the counter-attacking prey and the retreating predator, move in *ipsi-directional* formation (except when the mobbed lion predator refuses, to its disadvantage, to do so; e.g. Chevalier 2013).

The questions which were asked in this paper are first, whether such *ipsi-directional* alignment behaviours of giant honeybees at the nest surface with the flight path of preying wasps do have the power to drive the wasp out of the range of the bees’ nest, and second, whether the repelling capacity of shimmering flashes (Kastberger et al. 2008) is enhanced by this alignment behaviour. The data bring evidence for these surmises and document asymmetry in the mutual alignment responses of both counterparts (Fig. 5, S4/Online Resource 4) which benefits the bees to the disadvantage of the wasps. The findings support the *directed-shimmering-drives-wasps* hypothesis which expands upon the more general *defence hypothesis of shimmering* (Kastberger et al. 2008).

### Mechanisms behind directional alignment

Theoretically, if two parties move, each of them in random direction, correspondence of their headings is incidental (Fig. S3A-C/Online Resource 3). The opposite situation arises if significant portions of the moving paths of both parties are aligned, which may happen also independent from heading values. Basically, moving organisms may align themselves in their headings of their motions in a large variety of displays. In the simplest way, directional alignment is caused by vectored structures of the ambient milieu, such as acoustic or gravitation gradients, or a stream current which, for example, makes a trumpet fish swarm swaying back and forth with the wave action of the water (Kuiter 2001). A more self-determined way of individuals to align their posture or movement directions is observed when the presence of others triggers the heading of an entire group of specimens. This happens in fishes in schooling (Reynolds 1987) or in swimming together with larger specimens such as pilot fishes following sharks (Randall et al. 1997); it happens in bird flocks (e.g. Ballerini et al. 2008), in the alignment on pheromone trails (Wilson 1962; Dorigo et al. 2000) and, which is important here, also in the interaction between potential predators and their prey (Alcock 2005; Chevalier 2013). These latter forms of directional alignment are caused by interactions between usually moving agents which show reactive interventions, mutual signalling and individual decisions.

In this paper, we utilized the *latency* aspect to separate the *driving* cues from the *driven* responses in the alignment reactions. This method bases on the principle that any reaction or any decision produced upon a driving signal occurs with a typical delay after the onset of the respective stimulus. In a prey–predator interaction, however, this principle is more complex because both parties have a double role; they *emit* signals and also *respond* to stimuli of the counterpart while one of the goals, defence or predation, finally happen to dominate.

### Asymmetries between the directional alignments of the interacting counterparts

One of the covert principles behind directional correspondence, which has also been addressed in this paper, is the *symmetry* of its origin. Directional correspondence is evoked *symmetrically* if both counterparts align with similar strength and is evoked *asymmetrically* if only one of the two interacting counterparts has the ability to react, to decide and to align, or if one party dominates this kind of interaction. Theoretically, if neither the preying wasp nor the shimmering bees shows any tendency to mutual alignment, both counterparts would deliver a uniform distribution of their movement or posture directions in the entire circular range of 360°. The findings (Fig. 3), however, give evidence that directional alignment between shimmering bees and preying wasps is quite a dominant factor, and if the movements of both parties (*w* and *sh*) are compared in a stimulus–response paradigm by considering latencies, also asymmetries between the alignment responses of shimmering bees and wasps were traced (Fig. 5). By comparing the empirical data with the *movement heading model* (Fig. S3/Online Resource 3), it becomes clear that the phenomenon of *ipsi-directional dominance* (Figs. 3, 4, 5 and 6) can be seen as a form of steady-state alignment resulting from the continuous and directional interactions between prey and predator.

It is a matter of course that any alignment response between predators and their prey should be *asymmetric* due to the intrinsic difference of their goals. For the bees, as potential prey, any directional alignment will have more importance in benefitting the colony if the goal of alignment is to enhance the repellence power of shimmering (Kastberger et al. 2008). On the other side, wasps may recognize shimmering waves as a “nasty” visual cue to be “ignored” at the earliest possible moment. It is unlikely that any signal emitted by shimmering would benefit the wasps (cf. Kastberger et al. 2008), such as to prevent them from wasting time trying unsuccessfully to catch bees from the nest surface (which otherwise would be predicted by the *handicap principle*, Zahavi 1997). The findings brought up at least three aspects of *asymmetry* behind the alignment responses of shimmering bees and preying wasps, and, importantly, all of them are potentially benefitting the collective of honeybees.

### Three aspects of asymmetry

First, following roughly parallel courses, both counterparts were quite different in their turning velocities (Fig. 4e). When turning, shimmering waves were, independently of the heading of the wasps, more than four times as fast as the wasps; this makes shimmering bees to be more reactive than wasps on prey.

Second, the findings (Figs. 4–7; see also Movies 1–10/Online Resources 5–14) show that both counterparts may be aligned to each other at a level of up to 15 %, but with significant differences shimmering bees aligned their collective response with the wasp’s flight continuously for at least 100 ms (Figs. 5, S4/Online Resource 4) after the onset of the signals produced by the wasps’ flight manoeuvres. The motive force of the wasp for active alignment was here much lower, as it decreased substantially already 40 ms after the respective signal from the shimmering pattern (Fig. 5). These findings designate the shimmering bees as the more persisting and thus dominating part in the mutual alignment process.

Third, the bees aligned their shimmering waves with the same strength in all directions (Fig. 6e), with no directional preference with the flight path of the wasps. This means that the bees recognize the wasp as a threat independently of the direction of her flight path. The wasps, however, were more reactive to horizontal than vertical movements of shimmering (Fig. 7e). Their alignment reactions are obviously influenced by the topology of the bees’ nest site. This preference for horizontal structures can be seen as a form of ideomotor responses (which are otherwise known of being associated with psychological contexts in humans rather than with insect behaviours; see Carpenter 1852; Stock and Stock 2004).

In summary, the capacity for continuous directional alignment of the shimmering waves with the flight path of wasps enhances the repelling power of shimmering (Kastberger et al. 2008). The honeybees, as compared to predatory wasps, take at least a threefold advantage of an *asymmetry* in the prey–predator interaction: by a higher turning velocity of their moving patterns (Fig. 2e), by a greater persistence (Figs. 5, S4/Online Resource 4) and by a uniform directionality (Figs. 6e and 7e). These findings may support the surmise that directional alignment of shimmering giant honeybees towards a wasp on prey make up the phenomenon of *predator driving*. This phenomenon is generally associated with *mobbing* behaviour, such as in selfish herds of vertebrate species (Hamilton 1971; Curio 1978; Arnold 2000; Alcock 2005; Chevalier 2013), but is, until now, unknown in the insect world (cf. Tan et al. 2012).

## Electronic supplementary material

Below is the link to the electronic supplementary material.

Online Resource 1 Fig. S1 Experimental site and setup. (A, B) Back view of the hotel in Sauraha (Chitwan, Nepal) with the honeybee nest at the second floor; the cover of white linen protected the experimental site from sunlight, and guided the wasps from and to the relocated paper nest (positioned behind the yellow circles) but also to pass them along the experimental honeybee nest. (C) The experimental *Apis dorsata* nest (*nest*, blue arrow on the top left) is hidden behind the linen, HD-camera (vid, pink arrow), two cameras for stereoscopic imaging (v1-v2, orange arrows; Kastberger et al. 2011b, 2012, 2013b), laser vibrometer (lv, yellow arrow; Kastberger et al. 2013b), and infrared camera (ir, red arrow; Waddoup 2014); the cyan arrow (n2) marks a small second queen-less colony. (D) Close-up of the experimental *Apis dorsata* nest from the front; the wasp paper nest (E) was relocated behind it. (JPEG 19.2 MB)

Online Resource 2 Fig. S2 Definitions of angular relations between the movements of wasp (*w*) and shimmering (*sh*). (A) Angular deviation between the direction of flight of the wasp (θ_w_; red colour codes) and the direction of the shimmering wave (θ_sh_; blue colour codes) assessed in two subsequent frames (see Fig. 2). The directions θ_w_, θ_sh_ are related to eight angular sectors (cat θ = [1–8]). Equity in directions of *w*- and *sh*-movements is given by θ_w_ = θ_sh_. In the noted example, the direction of the shimmering wave was cat θ_sh_ = [8] and that of the wasp flight path was cat θ_w_= [7]. For the aspect of interaction *w → sh* (stimulus: *w*; responding party: *sh*), both directions result in a deviation of the shimmering wave from the wasp’s flight direction (as reference: θ_ref_ = θ_w_) with Δ cat θ_sh-w_ = cat θ_sh_ - cat θ_w_ = [+1]; for the reverse aspect (*sh → w*), the deviation was Δ cat θ_w-sh_ = cat θ_w_ - cat θ_sh_= [−1]. (B) *Ipsi-directional* alignment is displayed by the white sectors [+1],[0],[−1], *contra-directional* alignment by the black sectors [+3],[4],[−3],, the deviations Δ cat θ_ref_ = [+2] or [−2] represent here *intermediate* alignment. These definitions are applied for both aspects (*w → sh*: θ_ref_ = θ_w_; *sh → w*: θ_ref_ = θ_sh_) whereby *ipsi-directionality* is given by [cat θ_ref_ -1] ≤ Δ cat θ_w,sh_ ≥ [cat θ_ref_ +1] and *contra-directionality* by [cat θ_ref_ +4 -1] ≤ Δ cat θ_w,sh_ ≥ [cat θ_ref_ +4 +1]. (JPEG 2.52 MB)

Online Resource 3 Fig. S3 The headings of model participants w and sh in directional alignment. (A) Typical paths under headings ranging from *random-walk* (h_x_ = h_y_ = 0) to a straight walk with h_x_ = h_y_ = 0.50, where the movement components Δx, Δy were *positive* (directed to the right and upwards) in 75% of all cases (≡time steps), and *negative* (directed to the left and downwards) in the compliment of 25% of cases. (B) The rate histograms concerning the four categories of directional alignment (DIR_1–4_, cf. Figs. 6–7) in relation to the given magnitude of headings of the five sample paths, which were taken as equal for both movement components (h_x_= h_y_) of both model parties (h [w], h [sh]). The best match with the empirical data of Figs. 6–7 is achieved at h = +0.34 (see green-coded case). (C) Lookup table for the dependencies of the rates of DIR_1–4_ patterns from the headings in the model. (D) Determination of the best match between the empirical data as displayed in the Figs. 6–7 and the heading model. In this example, three headings were kept constant (h_x_[w] = +0.18, h_y_[w] = +0.34, h_x_[sh] = +0.24) while h_y_[sh] was varied (abscissa). The best match was here achieved at the maximum of the regression function (P[*χ* ^2^] = f (h_y_[sh]); R^2^ = 0.9828) at the ordinate value of P[*χ* ^2^] = 0. 968 corresponding with the abscissa value of h_y_[sh] = 0.17. (E) Best-match conditions (max P[*χ* ^2^]) between the empirical data (Figs. 6–7) and the model data; the green arrows refer to the headings at the best match under equity conditions (h [w] = h [sh]= 0.34; cf. panels A-B). (GIF 137 kb)

High Resolution Image (TIFF 3.04 MB)

Online Resource 4 Fig. S4 Supra-reference and sub-reference *ipsi-directional* dominance (D_id_) under time-shift conditions. (A) Graphical definitions under time-shift conditions with the difference between the rates of *ipsi- directional* and *contra-directional* alignment under synchrony conditions as the session-specific reference (ref D= ∆R_I-C_ [lag=0]): supra D_id_ ≡ ∆R_I-C_ [│lag│>0] > ref D; sub D_id_ ≡ ∆R_I-C_ [│lag│>0] < ref D. (B) Rates of supra-D_id_ values (R_1_ [supra D_id_]) which refer to the normalization with R_1_ [supra D_id_] + R_1_ [sub D_id_] = 1.0 per lag condition; e.g. the value R_1_ [supra D_id_] = 0.4 means that 40% of the sessions (n_ss_ = 50) show supra D_id_ under the respective lag, and 60% show the complimentary relation of sub-reference D_id_ . (C) Session-specific rates, given as means (full circles) and SEMs (vertical bars), regarding R_2_ [supra D_id_] and R_2_ [sub D_id_]; for explanation, the ordinate value R_2_ [supra D_id_] = +0.08 means that the rates of inter-frame intervals (cf. Fig. 5) signalling supra D_id_ are higher by 8% under the given lag than under synchrony conditions (lag =0); negative R_2_ [supra D_id_] values refer to the relative number of sub D_id_ cases. The results show that the R_2_ [supra D_id_] values of *w→ sh* were larger (P= 0.0020, *t*-test) than that of *sh → w* at time shifts of more than 40 ms (│lag│= 3-4). (JPEG 2.59 MB)

Online Resource 5 Movie 1 A free-flying wasp provokes shimmering waves at the surface of a giant honeybee (*Apis dorsata*) nest (recorded at a hotel site in Sauraha, Chitwan, Nepal). In the film, the wasp approaches the nest from the left side. A curtain of white linen was placed behind the bees’ nest (see Fig. S1/ Online Resource 1 and Methods) to urge the wasp to pass the bees’ nest when homing to or departing from the paper nest. The film documents 179 ff (≡3.58 s) showing the nest in a quiescent state before the wasp comes into the image at frame 52 (≡1.04 s; fps = 50 Hz). The incoming wasp provokes a wave propagating in up-right direction, which makes the wasp fly downwards and turn around. This initiates a second wave which propagates in up-left direction alike the wasp, which lastly disappears behind the linen curtain again at the left side of the image. (AVI 1.82 MB)

Online Resource 6 Movie 2 The same episode as in Movie 1/ Online Resource 5, but with the flight path of the wasp tracked: the big red full circle gives the momentary thoracic position of the wasp, the small red circles give the head position of the wasp in the 14 frames prior to the actual one, the fine yellow lines display the projected direction of the long body’s axis, and the thick pink connection line sketches the thoracic positions prior to the momentary position. The red text on the right bottom side refers to the relative time in ms of the presence of the wasp in the image; the time starts with 0 ms at the moment of the first appearance of the wasp. (AVI 1.84 MB)

Online Resource 7 Movie 3 The same episode as in Movie 2/ Online Resource 6 but only the moving objects (wasp and the shimmering bees) differential images are visualized (see Methods). The motion information is here segmented, so that differences in the original luminance between subsequent images (fps=50 Hz) with ∆ lum > 5 resulted in a white spot. The yellow line gives the contour of the nest. (AVI 821 kb)

Online Resource 8 Movie 4 The same episode as in Movie 3/ Online Resource 7, but without the tracking of the wasp’s flight, and the difference values of the original luminance between two subsequent images were not segmented by a threshold luminance value but coded by a rainbow scale with *blue* as low values and *red* as the largest differences. (AVI 1.26 MB)

Online Resource 9 Movie 5 The same episode as in Movie 4/ Online Resource 8, but with the tracking of the wasp’s flight. (AVI 1.35 MB)

Online Resource 10 Movie 6 The same episode as combined in the movies 1 & 4/ Online Resource 5–8, but the differential mode concerns here only the nest area. (AVI 2.29 MB)

Online Resource 11 Movie 7 The same episode as in Movie 6/ Online Resource 10, but with the tracking of the wasp’s flight. (AVI 2.28 MB)

Online Resource 12 Movie 8 The same episode as in movie 2/ Online Resource 5 but with slow motion (fps = 10 Hz, slow-motion factor = 2.0). (AVI 8.91 MB)

Online Resource 13 Movie 9 The same episode as in movie 5/ Online Resource 9 but with slow motion (fps = 10 Hz, slow-motion factor = 2.0). (AVI 8.80 MB)

Online Resource 14 Movie 10 The same episode as in movie 7/ Online Resource 11 but with slow motion (fps = 10 Hz, slow-motion factor = 2.0). (AVI 7.83 MB)
