# Supplementary figures and images for: Giant honeybees (Apis dorsata) mob wasps away from the nest by directed visual patterns
Source: Naturwissenschaften. 2014 Aug 29;101(11):861–73. doi: 10.1007/s00114-014-1220-0 (PMC4209238; doi:10.1007/s00114-014-1220-0)

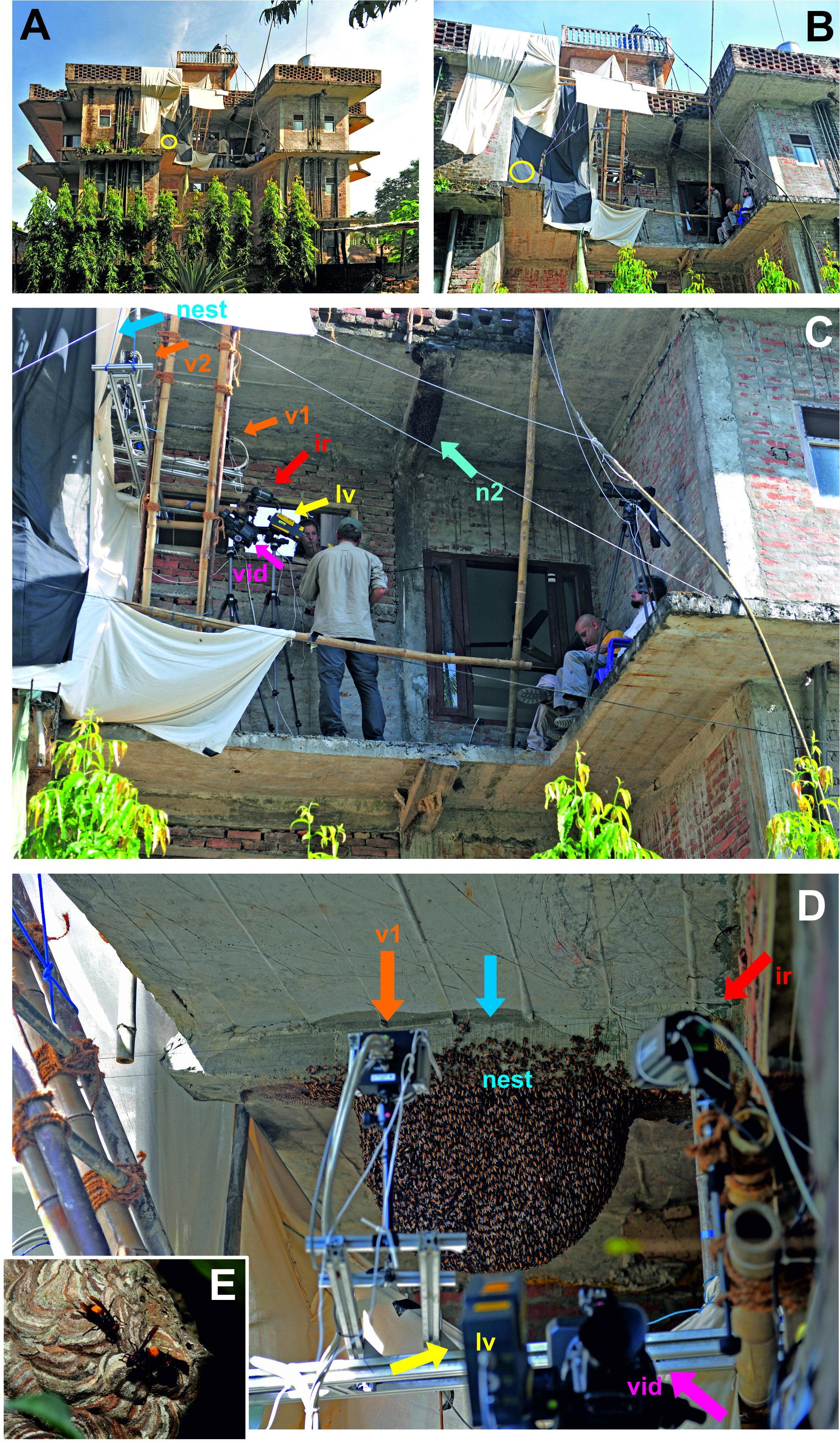

Supplement: Supplementary file 1 — Fig. S1 Experimental site and setup. (A, B) Back view of the hotel in Sauraha (Chitwan, Nepal) with the honeybee nest at the second floor; the cover of white linen protected the experimental site from sunlight, and guided the wasps from and to the relocated paper nest (positioned behind the yellow circles) but also to pass them along the experimental honeybee nest. (C) The experimental Apis dorsata nest (nest, blue arrow on the top left) is hidden behind the linen, HD-camera (vid, pink arrow), two cameras for stereoscopic imaging (v1-v2, orange arrows; Kastberger et al. 2011b, 2012, 2013b), laser vibrometer (lv, yellow arrow; Kastberger et al. 2013b), and infrared camera (ir, red arrow; Waddoup 2014); the cyan arrow (n2) marks a small second queen-less colony. (D) Close-up of the experimental Apis dorsata nest from the front; the wasp paper nest (E) was relocated behind it. (JPEG 19.2 MB) [file 114_2014_1220_Fig8_ESM.jpg]

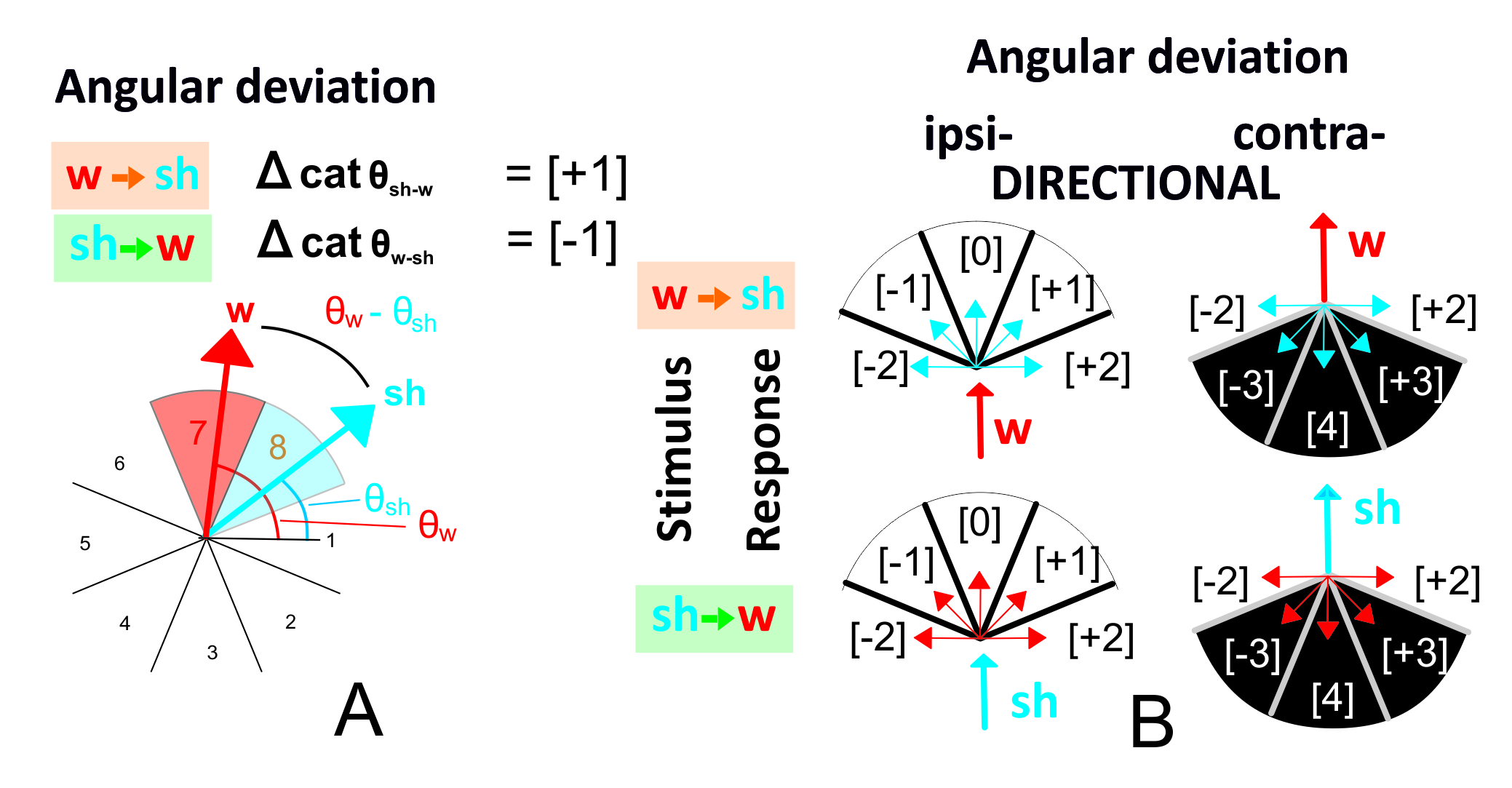

Supplement: Supplementary file 2 — Fig. S2 Definitions of angular relations between the movements of wasp (w) and shimmering (sh). (A) Angular deviation between the direction of flight of the wasp (θw; red colour codes) and the direction of the shimmering wave (θsh; blue colour codes) assessed in two subsequent frames (see Fig. 2). The directions θw, θsh are related to eight angular sectors (cat θ = [1–8]). Equity in directions of w- and sh-movements is given by θw = θsh. In the noted example, the direction of the shimmering wave was cat θsh = [8] and that of the wasp flight path was cat θw= [7]. For the aspect of interaction w → sh (stimulus: w; responding party: sh), both directions result in a deviation of the shimmering wave from the wasp’s flight direction (as reference: θref = θw) with Δ cat θsh-w = cat θsh - cat θw = [+1]; for the reverse aspect (sh → w), the deviation was Δ cat θw-sh = cat θw - cat θsh= [−1]. (B) Ipsi-directional alignment is displayed by the white sectors [+1],[0],[−1], contra-directional alignment by the black sectors [+3],[4],[−3],, the deviations Δ cat θref = [+2] or [−2] represent here intermediate alignment. These definitions are applied for both aspects (w → sh: θref = θw; sh → w: θref = θsh) whereby ipsi-directionality is given by [cat θref -1] ≤ Δ cat θw,sh ≥ [cat θref +1] and contra-directionality by [cat θref +4 -1] ≤ Δ cat θw,sh ≥ [cat θref +4 +1]. (JPEG 2.52 MB) [file 114_2014_1220_Fig9_ESM.jpg]

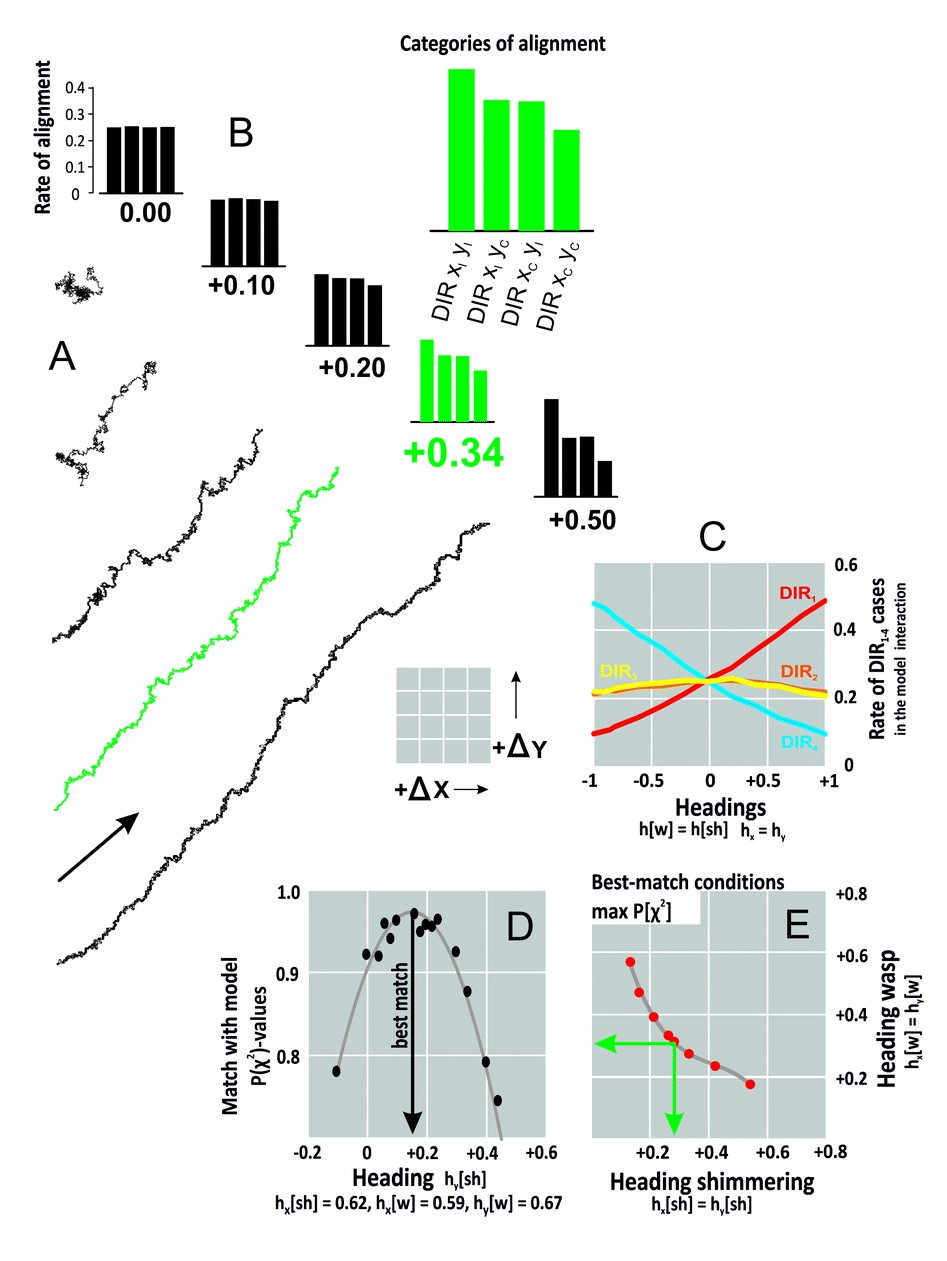

Supplement: Supplementary file 3 — Fig. S3 The headings of model participants w and sh in directional alignment. (A) Typical paths under headings ranging from random-walk (hx = hy = 0) to a straight walk with hx = hy = 0.50, where the movement components Δx, Δy were positive (directed to the right and upwards) in 75% of all cases (≡time steps), and negative (directed to the left and downwards) in the compliment of 25% of cases. (B) The rate histograms concerning the four categories of directional alignment (DIR1–4, cf. Figs. 6–7) in relation to the given magnitude of headings of the five sample paths, which were taken as equal for both movement components (hx= hy) of both model parties (h [w], h [sh]). The best match with the empirical data of Figs. 6–7 is achieved at h = +0.34 (see green-coded case). (C) Lookup table for the dependencies of the rates of DIR1–4 patterns from the headings in the model. (D) Determination of the best match between the empirical data as displayed in the Figs. 6–7 and the heading model. In this example, three headings were kept constant (hx[w] = +0.18, hy[w] = +0.34, hx[sh] = +0.24) while hy[sh] was varied (abscissa). The best match was here achieved at the maximum of the regression function (P[χ 2] = f (hy[sh]); R2 = 0.9828) at the ordinate value of P[χ 2] = 0. 968 corresponding with the abscissa value of hy[sh] = 0.17. (E) Best-match conditions (max P[χ 2]) between the empirical data (Figs. 6–7) and the model data; the green arrows refer to the headings at the best match under equity conditions (h [w] = h [sh]= 0.34; cf. panels A-B). (GIF 137 kb) [file 114_2014_1220_Fig10_ESM.gif]

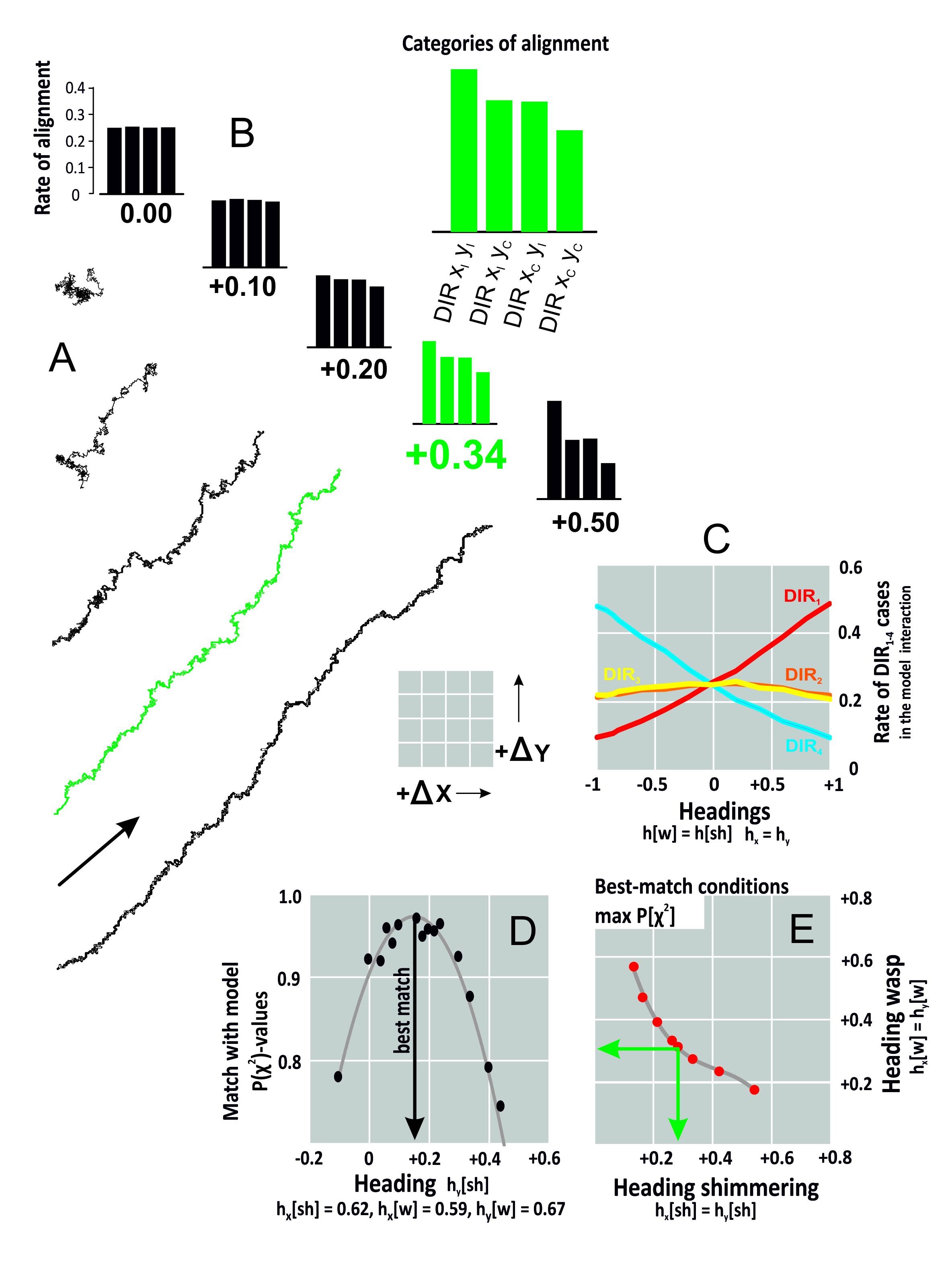

Supplement: Supplementary file 4 — High Resolution Image (TIFF 3.04 MB) [file 114_2014_1220_MOESM1_ESM.tif]

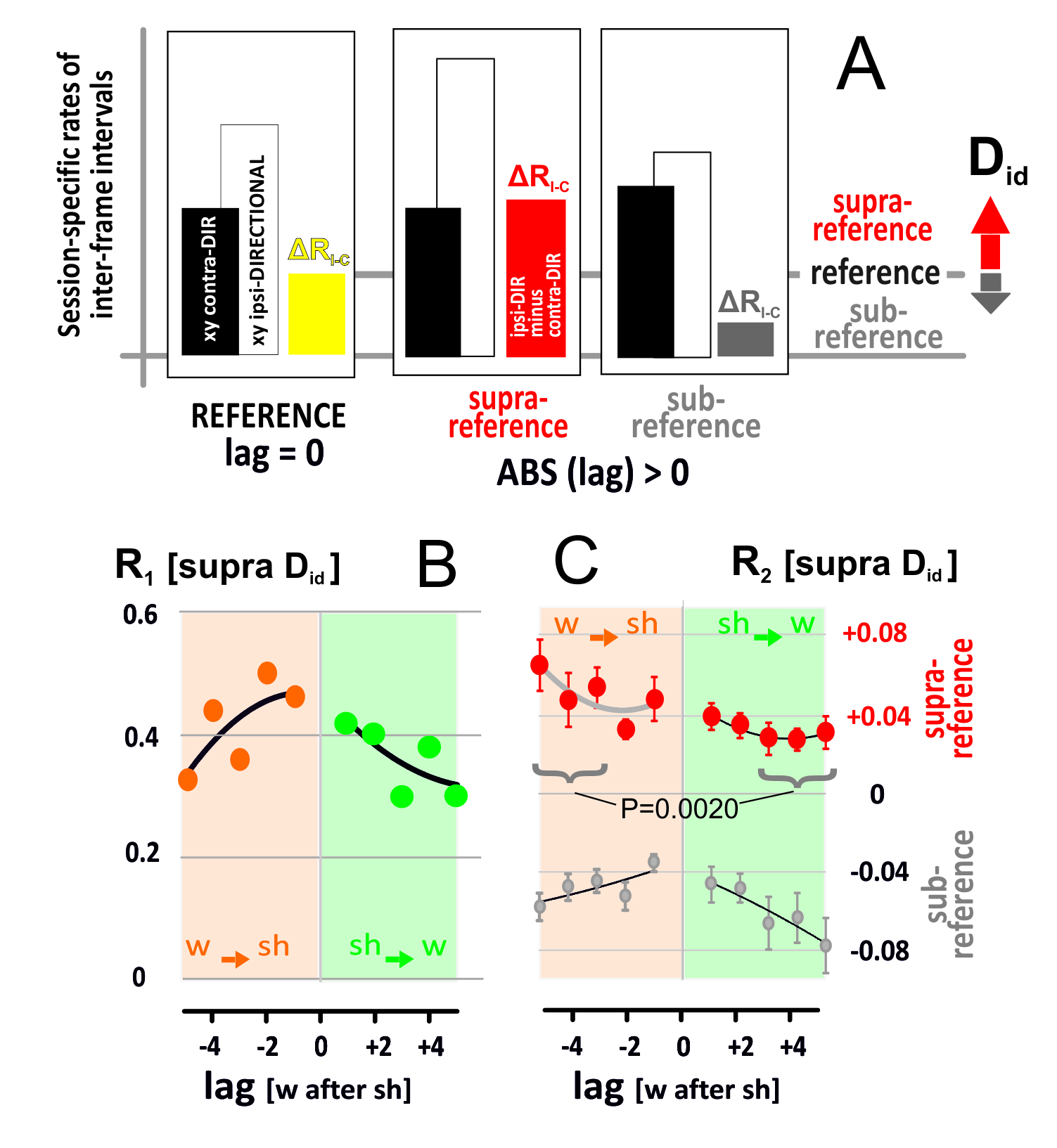

Supplement: Supplementary file 5 — Fig. S4 Supra-reference and sub-reference ipsi-directional dominance (Did) under time-shift conditions. (A) Graphical definitions under time-shift conditions with the difference between the rates of ipsi- directional and contra-directional alignment under synchrony conditions as the session-specific reference (ref D= ∆RI-C [lag=0]): supra Did ≡ ∆RI-C [│lag│>0] > ref D; sub Did ≡ ∆RI-C [│lag│>0] < ref D. (B) Rates of supra-Did values (R1 [supra Did]) which refer to the normalization with R1 [supra Did] + R1 [sub Did] = 1.0 per lag condition; e.g. the value R1 [supra Did] = 0.4 means that 40% of the sessions (nss = 50) show supra Did under the respective lag, and 60% show the complimentary relation of sub-reference Did . (C) Session-specific rates, given as means (full circles) and SEMs (vertical bars), regarding R2 [supra Did] and R2 [sub Did]; for explanation, the ordinate value R2 [supra Did] = +0.08 means that the rates of inter-frame intervals (cf. Fig. 5) signalling supra Did are higher by 8% under the given lag than under synchrony conditions (lag =0); negative R2 [supra Did] values refer to the relative number of sub Did cases. The results show that the R2 [supra Did] values of w→ sh were larger (P= 0.0020, t-test) than that of sh → w at time shifts of more than 40 ms (│lag│= 3-4). (JPEG 2.59 MB) [file 114_2014_1220_Fig11_ESM.jpg]
